# A Novel Ventilator Design for COVID-19 and Resource-Limited Settings

**DOI:** 10.3389/fmedt.2021.707826

**Published:** 2021-10-04

**Authors:** Michael Madekurozwa, Willy V. Bonneuil, Jennifer Frattolin, Daniel J. Watson, Axel C. Moore, Molly M. Stevens, James Moore, Jakob Mathiszig-Lee, Joseph van Batenburg-Sherwood

**Affiliations:** ^1^Department of Bioengineering, Imperial College London, London, United Kingdom; ^2^Department of Materials, Department of Bioengineering and Institute of Biomedical Engineering, Imperial College London, London, United Kingdom; ^3^Department of Surgery and Cancer, Imperial College London, London, United Kingdom; ^4^Department of Anaesthetics and Perioperative Medicine, Royal Marsden Hospital, London, United Kingdom

**Keywords:** ventilator, supply chain, COVID-19, respiratory disease, intensive care, medical device

## Abstract

There has existed a severe ventilator deficit in much of the world for many years, due in part to the high cost and complexity of traditional ICU ventilators. This was highlighted and exacerbated by the emergence of the COVID-19 pandemic, during which the increase in ventilator production rapidly overran the global supply chains for components. In response, we propose a new approach to ventilator design that meets the performance requirements for COVID-19 patients, while using components that minimise interference with the existing ventilator supply chains. The majority of current ventilator designs use proportional valves and flow sensors, which remain in short supply over a year into the pandemic. In the proposed design, the core components are on-off valves. Unlike proportional valves, on-off valves are widely available, but accurate control of ventilation using on-off valves is not straightforward. Our proposed solution combines four on-off valves, a two-litre reservoir, an oxygen sensor and two pressure sensors. Benchtop testing of a prototype was performed with a commercially available flow analyser and test lungs. We investigated the accuracy and precision of the prototype using both compressed gas supplies and a portable oxygen concentrator, and demonstrated the long-term durability over 15 days. The precision and accuracy of ventilation parameters were within the ranges specified in international guidelines in all tests. A numerical model of the system was developed and validated against experimental data. The model was used to determine usable ranges of valve flow coefficients to increase supply chain flexibility. This new design provides the performance necessary for the majority of patients that require ventilation. Applications include COVID-19 as well as pneumonia, influenza, and tuberculosis, which remain major causes of mortality in low and middle income countries. The robustness, energy efficiency, ease of maintenance, price and availability of on-off valves are all advantageous over proportional valves. As a result, the proposed ventilator design will cost significantly less to manufacture and maintain than current market designs and has the potential to increase global ventilator availability.

## Introduction

During the COVID-19 pandemic, one of the first challenges to healthcare systems worldwide was the supply of intensive care unit (ICU) ventilators. In the early days of the pandemic, ~30% of hospitalised patients required invasive ventilation (1). This stressed existing ventilator stocks in countries hit with early waves of the disease, prompting calls for increased production of existing designs. The ratio of citizens per ventilator provided a first order estimate of the number of ventilators that might be required, leading to an initial estimate of 880,000 new ventilators worldwide. In 2019, global ventilator production was estimated at 77,000 per year (2).

As stories emerged that clinicians were having to make the difficult choice of which patients received ventilation, designers and other manufacturers were called upon to produce alternative designs that could be used as ventilators (1, 3). Governments across the world encouraged engineers, clinicians, scientists, and the broader public to develop emergency use ventilators. Normally, ICU ventilators would be required to meet standards equivalent to ISO 80601-2-12 for approval in the EU and other large markets. However, in the UK, the Medicines and Healthcare Products Regulatory Agency (MHRA) provided special performance criteria that could alternatively be met for “emergency use” designs. The USA's Food and Drug Administration (FDA) also began approving emergency use ventilators under reduced criteria.

Since the 1952 polio pandemic, mechanical ventilation has been a cornerstone of intensive care medicine (4). It is required when a patient is unable to meet their oxygen requirements with natural breathing. Ventilators supplement the patient's respiration by delivering pressurised air or oxygen-enriched gas, thereby increasing the amount of oxygen delivered in each breath. Ventilation also helps to clear the excess carbon dioxide that builds up in the blood, which is important to prevent the narcosis and acidosis associated with carbon dioxide accumulation (5).

The first ventilators were manual, similar to the disposable Bag Valve Masks (BVM) that paramedics use to provide short term, manually-powered ventilation in modern ambulances (4). In modern ventilators, inspiratory flow is typically achieved using pressurised gas supplies regulated with proportional control valves or with a mechanism such as bellows to pressurise the gas (6). This pressure reduces or eliminates the required respiratory effort. Additionally, the positive pressure used to drive the gases into the lungs can open collapsed areas of the lungs, increasing the surface area available for gas exchange. The expiratory phase of ventilation provides carbon dioxide clearance, which is required to prevent the acidifying effect of CO_2_ on blood and its sedating effect on the central nervous system. Expiratory flow is driven by the pressure differential between the lungs and the pressure at the patient connector, which is controlled actively or passively by the ventilator (6).

Ventilation can be divided into invasive and non-invasive methods. In Non-Invasive Ventilation (NIV), pressurised gas is administered *via* a tight-fitting face mask, nasal tubes or a specially designed helmet. In contrast, invasive ventilation requires sedation of the patient and the insertion of an endotracheal tube (ETT) or a tracheostomy, a procedure that requires trained professionals. This makes NIV an attractive option for healthcare providers, particularly when equipment and staff are in short supply.

The two most common forms of NIV are Continuous Positive Airway pressure (CPAP), which provides a single pressure to aid oxygenation, and Bi-Level Positive Airway Pressure (BiPAP), which provides an additional high pressure phase during inhalation to aid ventilation. NIV requires a gas-tight fit for the mask in order to maintain positive pressure and limit excessive consumption of compressed gas. This is particularly important in the context of respiratory viruses, as leakage of pressurised gas around the mouth risks aerosolisation and distribution of the virus, which can endanger healthcare staff (7). NIV was found to reduce the numbers of patients that progress to invasive ventilation, but by varying amounts across studies, such as 20% (8), 38% (9), and 58% (10). Given the demand for intensive care beds during peaks of the pandemic, along with evidence of improved outcomes in other conditions (11), NIV is a key part of the clinical guidance for treating COVID-19 in the UK (12).

Invasive ventilation requires the administration of an anaesthetic and the insertion of an ETT into the trachea, *via* the mouth. If a patient is expected to remain ventilated for more than a week, this is often converted to a tracheostomy, where a shorter tube is inserted *via* the front of the neck which allows for reduced sedation and the gradual weaning of ventilatory support. Whilst there are significant risks associated with invasive ventilation, it is necessary for patients with severe respiratory failure. The technical challenge with invasive ventilation is to provide the required assistance without over-pressuring the lungs, which would lead to barotrauma (13). This task is made more challenging by the fact that the compliance and flow resistance of the lungs change as the condition of the patient improves or deteriorates.

There is a wide range of invasive ventilation modes available, [for reviews see e.g., (14, 15)]. The most common ones are Volume Controlled Ventilation (VCV), Pressure Controlled Ventilation (PCV) and Pressure Regulated Volume Control (PRVC). The typical form of VCV is the most basic invasive ventilatory mode and delivers a fixed volume, termed Tidal Volume, at a fixed flow rate. The benefit of this approach is that the Minute Volume (Tidal Volume multiplied by Respiratory Rate) is maintained irrespective of changes in lung compliance or airway resistance. However, without safeguards the peak inspiratory pressure can rapidly increase to hazardous levels if lung compliance reduces, potentially leading to barotrauma (6).

In PCV, the maximum inspiratory pressure is set, rather than the Tidal Volume. This mode protects the patient from barotrauma by limiting the peak pressure (16). Additionally, by delivering an approximately constant pressure throughout inspiration, alveolar distension is maintained throughout the inhalation and the peak airway pressure is lower compared to VCV. However, PCV requires more frequent clinical input, with the peak inspiratory pressure needing regular adjustment in order to maintain the desired Minute Volume whilst avoiding hypoventilation, overdistension or volutrauma (6).

Alarm systems on modern ventilators mitigate some of the downsides of the more basic modes, for example with Tidal Volume alarms and hard limits on peak pressure. However, the workload demands for medical staff operating ventilators remain high (17) and hence modern modes aim to reduce this. PRVC combines the two basic forms of ventilation with the aim of delivering the advantages of both, whilst eliminating their shortcomings. In this mode, the ventilator aims to achieve a desired Tidal Volume by varying the pressure delivered during inspiration, with a maximum value set by the operator. This ideally results in a situation where pressure is kept to a minimum and where changes in patient lung mechanics do not alter the Minute Volume delivered (as long as safety limits are not violated). The implementation of PRVC within ICUs has been associated with a significant improvement in the proportion of patients ventilated in a manner that avoids lung injury (18, 19).

For all ventilation modes, exhalation occurs due to lung recoil after inhalation. If the lungs returned to atmospheric pressure, the alveoli could collapse. This would result in a cyclic reopening of the lungs on each respiratory cycle that would increase inflammation and require higher ventilation pressures. To prevent this, ventilators maintain a Positive End Expiratory Pressure (PEEP), which prevents de-recruitment of alveoli and maximises the surface area available for gas exchange. In simple devices, such as BVMs, PEEP can be set and adjusted using a spring-loaded diaphragm on the expiratory port. While cheap to employ, this method results in a fixed expiratory resistance and increases the time needed to expire. In contrast, modern ICU ventilators often use electronic valves to regulate the expiratory resistance in order to achieve the desired PEEP (6).

In summary, mechanical ventilation requires a device that can control volume or pressure during inhalation, and the pressure at the end of exhalation. A large range of designs for Emergency Ventilators were produced globally in response to the COVID-19 pandemic. These ranged from actuated BVMs to simplified versions of existing anaesthetic or ICU ventilators. For our contribution, we established these guiding design principles.

*Principle 1: minimise supply chain overlap with existing ventilator designs*. As existing regulatory approved ventilator designs were preferred, a new ventilator design should minimise overlap with the existing supply chain as much as possible. Certain components such as pressure and oxygen sensors cannot be safely avoided, but the mass flow controllers, or proportional solenoid valves and flow meters typically used to regulate compressed gas flows, were in extremely short supply.

*Principle 2: design for supply chain flexibility and straightforward manufacturing*. The need for new ventilator designs was global, so a design that did not depend on specific supply chains could have a broader impact. Therefore, the system should use widely available components and minimise dependence on specific components or complex machined parts. Design simplicity also reduces supply chain dependence by reducing the number of potential sources of component bottlenecks.

*Principle 3: design for mechanical robustness*. Achieving continuous ventilation for days to weeks requires high levels of mechanical robustness. Moving parts are prone to wear and are the most likely source of failure. The design should therefore minimise the number of moving parts, allow for simple repair or replacement of worn components, and utilise components that can be safely operated in an oxygen-rich environment.

*Principle 4: accuracy, repeatability and usability*. The design should be able to operate as a full ICU ventilator, to provide long-term, precise ventilation compliant with regulatory performance standards. Due to the scarcity of highly trained operators, the system should be straightforward to use with minimal clinical workload.

To address these design criteria, we developed a new way to achieve ICU-level ventilation that could be built with widely available, off-the-shelf components. In particular, the proposed design uses on-off solenoid valves, which are low-cost, low power-consumption, easy to maintain and repair and widely available globally. This paper describes the design concept and rigorous testing of the accuracy, precision and durability of our prototype.

## Materials and Methods

### Design Principle

**Figure 1:**
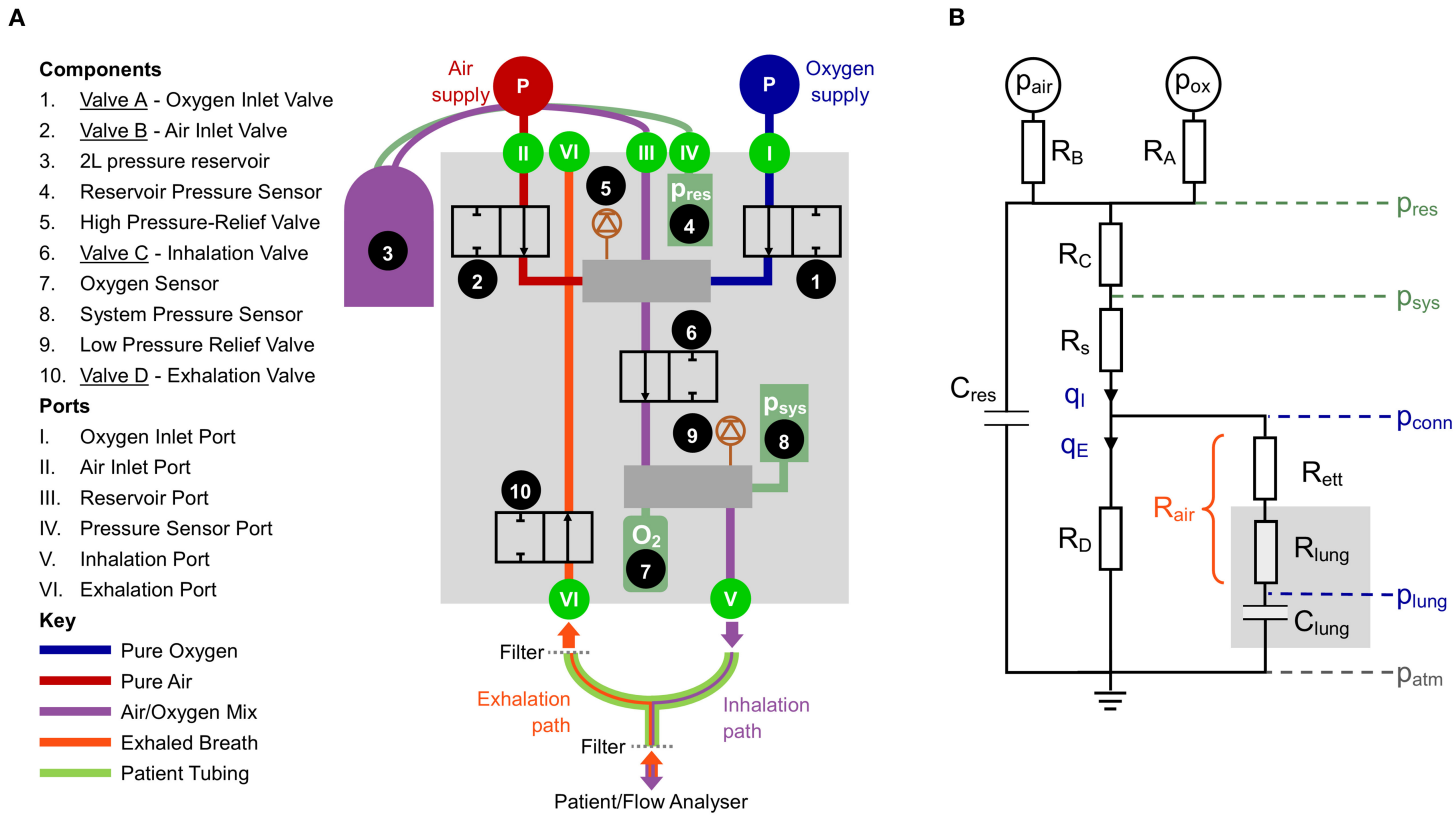
**(A)** Schematic of the proposed design. **(B)** Simplified lumped parameter model of the system. Pressures: reservoir pressure (p_res_), system pressure (p*_sys_*), pressure at the patient connector (p_conn_), pressure in the distal lungs (p_lung_). Green indicates measured and blue indicates calculated. Flow rates: q_I_, inhaled flow, q_E_, exhaled flow. The resistances represent combined influence of multiple components: R_A_, oxygen port connections and Valve A; R_B_, air port connections and Valve B; R_C_ pneumatic pathways between the reservoir and downstream manifold; R_D_, all pneumatic components in the exhalation pathway; R_s_, pneumatic components downstream of p_sys_; R_ett_, endotracheal tube; R_lung_, resistance in the lung, predominantly the upper airways; R_air_, combination of R_ett_ and R_lung_, used to estimate p_lung_.

The critical feature of our proposed solution to the design requirements is replacing the proportional solenoid valves found in traditional ventilators with 2/2 (two-port), or “on-off,” solenoid valves (addressing Design Principles 1-3). Depending on the voltage applied across them, these valves operate in a “binary” mode to either prevent flow (closed) or allow flow (open). The flow rate in the open position is predominantly dependent on the flow coefficient, *K*_*v*_, and the pressure drop across the valve. The design uses four on-off solenoid valves that we label A-D (Figure 1A).

Valves A and B (Components 1 and 2) are used to control the mix of oxygen and air, respectively. Rather than providing continuous flow, as would be done with proportional valves, on-off valves provide step changes in flow resistance that require damping before gas is delivered to the patient. This is accomplished by charging a fixed volume (2 L) reservoir with the appropriate gas mixture (Component 3). During the exhalation phase, a breath of predefined volume is stored up in the reservoir. By regulating the time that Valves A and B are open (in sequence), it is possible to control both the Tidal Volume (V_T_) and the oxygen concentration of each breath, based on the ideal gas law.

At the beginning of an inhalation, Valve C (Component 6) opens, allowing pressurised gas from the reservoir to be delivered to the patient. Valve C closes once the desired Tidal Volume has been delivered. The oxygen concentration of the delivered gas is monitored with an oxygen sensor (Component 7). Exhalation starts with Valve D (Component 10) opening and ends when the pressure in the lungs declines to the target PEEP set by the user. Components 5 and 9 are mechanical pressure relief valves that add an additional level of safety.

#### System Dynamics

Accurately achieving the desired system dynamics requires characterisation of the system components. Parameters such as the reservoir volume and *K*_*v*_ of the valves and other resistive elements govern the pressure distribution throughout the system. This part of the design process was aided by a lumped parameter representation, shown in Figure 1B.

The resistances *R*_*A*_ and *R*_*B*_ (given by $R=q/K_{v}^{2}$) and the pressure drop between *p*_*air*_ or *p*_*ox*_ and *p*_*res*_ determine the flow rate, *q*, from each gas supply into the reservoir (all pressures notated as *p* are given as absolute). The reservoir pressure is directly measured relative to the atmosphere (*p*_*res*_ − *p*_*atm*_) using a sensor coupled to the reservoir. The gas in the reservoir can be modelled using the ideal gas law and provides a compliance, *C*_*res*_ = *V*_*res*_/*p*_*res*_. The flow-pressure relationship of the pathway from the reservoir to the pressure sensor (*p*_*sys*_) attached to the downstream manifold (represented by the resistance *R*_*C*_ in Figure 1B) can be characterised using a power law:


(1) $$\begin{array}{c} q_{I}=a_{I}{\left(p_{res}-p_{sys}\right)}^{n_{I}} \end{array}$$

for which the constants *a*_*I*_ and *n*_*I*_ can be calculated by calibration and $R_{C}=\frac{1}{a_{I}}{\left(p_{res}-p_{sys}\right)}^{1-n_{I}}$. The flow rate *q*_*I*_ is the flow rate delivered to the patient, and thus monitoring *p*_*res*_ and *p*_*sys*_ negates the need for a flow sensor, simplifying the system in line with Design Principles 1 and 2. Sample calibration data for Equation 1 is shown in Supplementary Figure S1A.

During inhalation, there is an additional pressure drop between *p*_*sys*_ and the patient connector due to the pneumatic connections within the ventilator, with resistance denoted *R*_*s*_. The pressure at the patient connector can be calculated according to


(2) $$\begin{array}{c} p_{conn}=p_{sys}-{\left(\frac{q_{I}}{K_{v,s}}\right)}^{2} \end{array}$$

where *K*_*v,s*_ is the flow coefficient of the components comprising *R*_*s*_ and can be calibrated (see Supplementary Figure S1A). From the patient connector, the gas flows across a heat and moisture exchange (HME) filter and an ETT (combined resistance notated *R*_*ett*_). The airway resistance *R*_*lung*_ is dominated by the first 5–10 generations of branches in the lungs (20), and is thus modelled upstream of the lung compliance, *C*_*lung*_, which represents the expansion characteristics of the distal airways, predominantly the alveoli. For brevity, the combination of *R*_*ett*_ and *R*_*lung*_ can be considered as the total airway resistance, *R*_*air*_ and the pressure in the lungs during inhalation can be calculated according to


(3) $$\begin{array}{c} p_{lung}=p_{conn}-R_{air}\;q_{I} \end{array}$$

Note that while the resistance of the lungs is considered to be relatively independent of flow rate, flow through the ETT is turbulent and hence *R*_*air*_ = *f*(*q*). During exhalation, the lung pressure is given by


(4) $$\begin{array}{c} p_{lung}=p_{sys}+R_{air}\;q_{E} \end{array}$$

The directly measured pressure, *p*_*sys*_, can be used in Equation 4 because no flow passes through *R*_*s*_ during exhalation, thus *p*_*sys*_ = *p*_*conn*_.

The exhaled flow *q*_*E*_ passes through the resistance of the exhalation pathway (*R*_*D*_ in Figure 1B) comprising an additional filter on the exhalation side of the ventilator, Valve D and the internal pneumatic connections of the exhalation pathway. These components can be lumped into a single pressure-flow relationship according to


(5) $$\begin{array}{c} q_{E}=a_{E}{\left(p_{sys}-p_{atm}\right)}^{n_{E}} \end{array}$$

for which the constants *a*_*E*_ and *n*_*E*_ can be calculated by calibration (Supplementary Figure S1B).

The compliance of the gas in the components external to the patient is not included in the model as it can be estimated to be *V*_*tube*_/*p*_*atm*_ ~ 0.6 *ml*/*cmH*_2_*O*, which is 1-2 orders of magnitude smaller than the compliance of a normal or pathologic human lung.

#### Operation

Our prototype was developed to predominantly operate in PRVC mode (consistent with Design Principle 4), in which the clinician sets Tidal Volume (V_T_), Respiratory Rate (RR), PEEP, the proportion of inspiration to expiration time (I:E ratio) and the fraction of inspired oxygen (FiO_2_).

**Figure 2:**
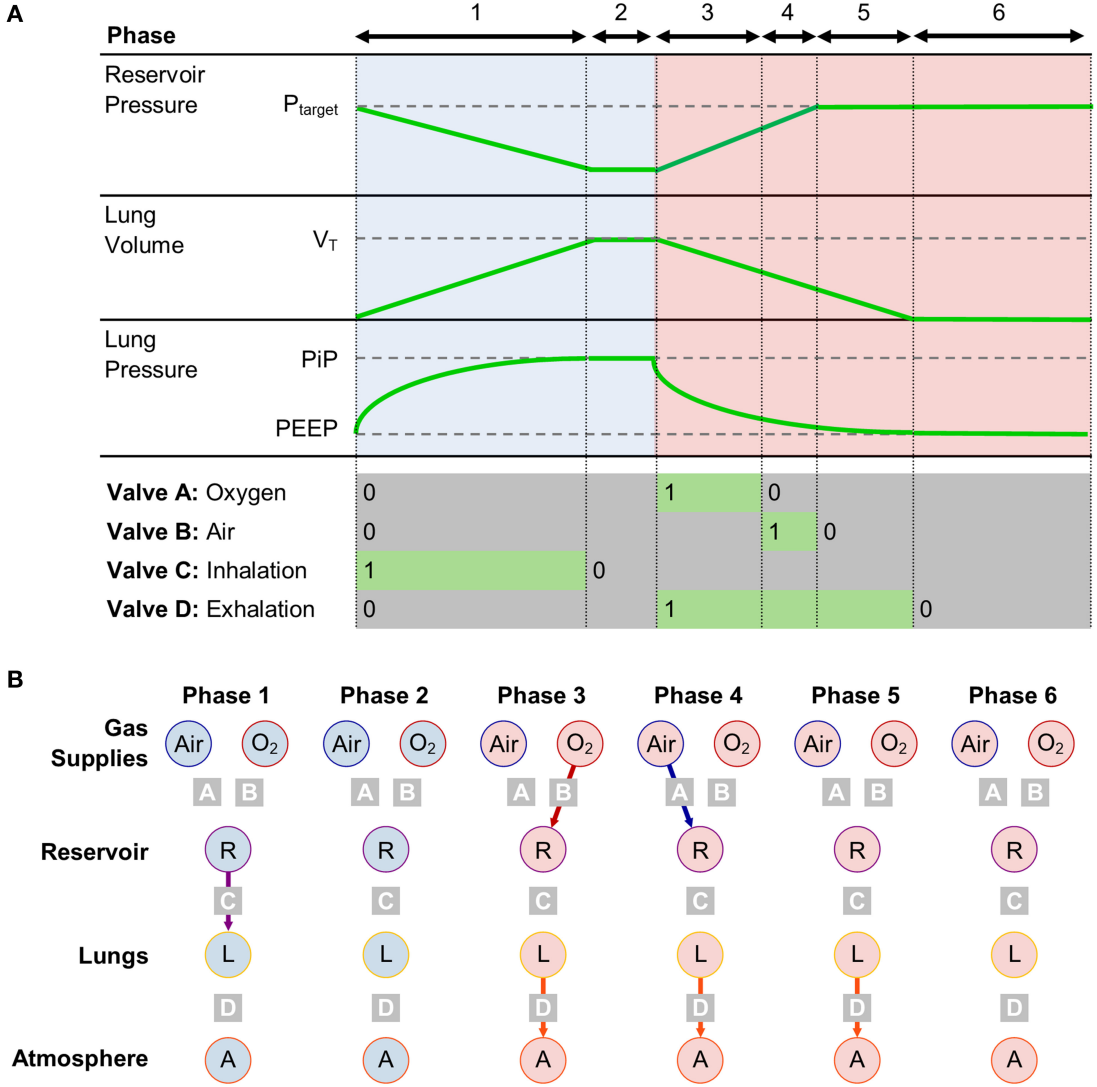
**(A)** Timing diagram for operation in PRVC mode, separated into 6 phases, with corresponding valve states and key system parameters. **(B)** Compartment model showing connections between gas storing components in each phase.

Inhalation starts with all valves closed. Valve C then opens (Phase 1), allowing the pre-pressurised gas mixture in the reservoir to flow into the lungs. When the delivered volume reaches *V*_*T*_, Valve C closes (Figure 2). The system control algorithm aims to minimise Phase 2, such that *V*_*T*_ is delivered exactly in the desired time, *T*_*in*_ (which is given by *T*_*in*_ = 60/*RR* × 1/(1 + *E*), where *E* is taken from the I:E ratio, and by convention *I* = 1). The exhalation starts (Phase 3) with Valve D opening at *T*_*in*_ in order to maintain consistency with the I:E ratio and RR. The ideal behaviour of Valve D is to close when *p*_*lung*_ reaches PEEP. The value of *p*_*lung*_ can be calculated according to Equation 4, based on an estimate of *R*_*air*_, calculated from the flow and pressure waveforms during exhalation. When Valve D closes, the system enters Phase 6, during which the pressure in the lung remains at PEEP. During the exhalation, the upstream side of the system prepares the next breath by opening first Valve A (Phase 3), then Valve B (Phase 4) until *p*_*res*_ reaches the target value determined by the control algorithm.

With this approach, the ventilator control problem reduces to a simple timing control, which can be achieved with minimal computing power and complexity.

### Experimental Setup

#### Prototype Components

The prototype system was built using off-the-shelf components. A National Instruments data acquisition card (DAQ, NI 6002) was run using custom LabVIEW code from a standard PC. A programmable logic controller (Barth STG600) was used as a multi-channel relay to control the valve switching and as a watchdog for the DAQ. The solenoid valves came from Emerson (262 series), with 1.2 mm orifices (Valves A and B), 2.4 mm orifice (Valve C) and 5.6 mm orifice (Valve D). Valve D was a normally closed (NC) valve, but for safety the final design would use a normally open (NO) valve. The pressure sensors were from Omegadyne (PXM319), and the oxygen sensor was from Teledyne (R-22MED). Pneumatic fittings (manifolds, connectors and bulkheads) were all standard off-the-shelf components using 1/8″-1/2″ BSP threads or 10 mm push fittings, with the exception of the ventilator tubing connection ports, which were machined from brass. Two duplicate builds of the prototype were used in the testing process.

#### Flow Analyser and Test Lungs

To test the accuracy of the prototype, a commercially available ventilator flow analyser was used. The Citrex H5 (IMT analytics) has bi-directional flow sensing, as well as pressure and oxygen measurement (accuracies ±0.1 l/min, ±0.1 mbar and ±1% O_2_, respectively) and was placed downstream of the patient Y-connector (see Figure 1A). Commercially available test lungs, the SmartLung and EasyLung (IMT analytics), were used for all tests. The SmartLung has adjustable compliances rated at 10, 15, 20, and 30 ml/cmH_2_O, and resistances rated at 5, 20, 50, and 200 cmH_2_O/(l/s). The EasyLung has a set compliance rated at 25 ml/cmH_2_O and a resistance rated at 20 cmH_2_O/(l/s). In the interest of brevity, we introduce the terminology RX/CY to indicate a test lung configuration with resistance X and compliance Y.

While these stated ratings are broadly indicative of the test lung performance overall, instantaneous values of resistance can vary widely. The resistance in the test lung, equivalent to *R*_*air*_, represents both the ETT (which has flow-rate dependent or “parabolic” resistance) and the patient lungs (which have constant or “linear” resistance). The relative contribution of each varies with ventilation and lung conditions and hence a single characteristic resistance can never be wholly representative of all conditions. The EasyLung and SmartLung use an orifice-plate resistor design, which generates a “parabolic” resistance, with the rated value typically reported at 30 or 60 l/min. For completeness, we characterised the resistance-flow rate characteristics of the test lungs (Supplementary Figure S2).

The compliance of test lungs can be both volume- and rate-dependent, so is less easily characterised. Of particular relevance is the maximum volume at which the test lungs can be operated. Both the EasyLung and SmartLung are silicone bags in a leaf-spring casing. Under normal circumstances, the latter determines the compliance, but when the volume is exceeded, further expansion requires deformation of the silicone bag, which is significantly less compliant. This limits the theoretical usable pressure to *V*_0_/*C*−*PEEP* (where *V*_0_ is the bag volume). However, the actual maximum pressure is lower as *V*_0_ is reduced by the method of varying compliance (a sliding bar that reduces the length of the leaf spring). The result of exceeding the maximum volume can be seen in the traces in Supplementary Figure S7, where the pressure rapidly increases at the end of the inhalation.

### Experimental Testing

To evaluate the efficacy of the design, we conducted a series of benchtop tests. Firstly, to assess the accuracy and precision of the prototype, a parametric sweep of ventilation parameters was carried out with a sample test lung configuration. Secondly, to test robustness to different lung conditions and pressure supplies, the testing regime required by ISO 80601-2-12:2020 was conducted in triplicate, using both standard 4-bar pressure gas supplies and a home-use oxygen concentrator. Finally, the durability of the prototype system was evaluated by continuously ventilating for 15 days.

#### Parametric Sweep

A parametric sweep of different combinations of clinical input parameters was conducted (Table 1) using an R20/C20 test lung configuration, which represents a clinically plausible case of severe respiratory disease. This data enabled (i) comparison between internal measurements from the prototype and the flow analyser and (ii) evaluation of the ability of the system to achieve parameter targets. The parametric sweep was automated, with simultaneous parameter changes applied every 15 breaths. The last 9 breaths were extracted for analysis, after allowing 6 breaths for the system to transition between different parameter inputs.

**Table 1:** Range of input parameters tested for the ventilation parametric sweep.

**Clinical parameter**	**Value**
V_T_ (ml)	200, 400, 600
RR (bpm)	10, 20, 35
PEEP (cmH_2_O)	5, 10, 15
I:E ratio	1:1, 1:2, 1:4
FiO_2_ (%)	60, 75, 90

*All possible combinations were tested, provided that p_peak_ was <45 cmH_2_O*.

Parametric evaluation of ventilation performance is complicated by the fact that certain combinations of ventilation parameters and lung characteristics would not be used clinically (as they would result in dangerously high patient pressures) and that the test lungs are only designed to operate within certain ranges. The test lung volume is 1,000 ml, above which the compliance decreases rapidly. For the compliance used for parametric testing (20 ml/cmH_2_O), the test lung would reach its maximum expansion at 50 cmH_2_O—PEEP. For an ideal test lung (constant *C*_*lung*_ and *R*_*air*_), the peak pressure for a given set of ventilation parameters can be estimated according to


(6) $$\begin{array}{c} PiP=PEEP+\frac{V_{T}}{C_{lung}}+R_{air}\bar{q} \end{array}$$

where PiP is the peak inspiratory pressure (gauge pressure) and the average flow rate is estimated according to $q=V_{T}/T_{ins}.$ We used Equation 6 to dismiss any cases where PiP was expected to exceed 45 cmH_2_O, as well as setting this as the peak pressure for our alarm system (which, when triggered, closes Valve C and opens Valve D to rapidly reduce lung pressure). The accuracy of Equation 6 is dependent on the waveform characteristics, which are influenced by the non-ideal behaviour of the test lungs (Section: Flow Analyser and Test Lungs). The peak pressure was exceeded for some cases, and these were eliminated from further evaluation. Overall, 166 cases out of a possible 243 cases, were analysed. A complete list of parameters included in the final analysis is compiled in Supplementary Table S1.

From the prototype, pressures, flow rates, and valve status (open/closed) were recorded at a frequency of 200 Hz. Four parameters were calculated from the acquired traces: RR, FiO_2_, achieved PEEP and V_T_. RR was calculated as the reciprocal of the time between two consecutive rising edges on the Valve C signal. FiO_2_ was calculated as the average measurement from the oxygen sensor during each exhalation. PEEP was calculated by taking the average pressure during the last 50 ms of the exhalation phase of each breath, as defined by ISO 80601-2-12:2020. V_T_ was calculated by integrating the inhaled flow rate, using trapezoidal integration. Values of inhaled and exhaled volume, respiration rate (RR), I:E ratio, FiO_2_ and PEEP were recorded with the flow analyser for comparison. A Bland-Altman analysis was carried out to investigate where there were systematic and/or random differences between prototype and flow analyser outputs.

#### ISO 80601-2-12 Testing

Seven tests were conducted according to the conditions outlined in ISO 80601-2-12:2020 (Table 2) along with a default initial condition. All tests were started at the default settings, with the FiO_2_ set to match that of the relevant test. Parameter changes were applied according to the values in the table, and 40 breaths of data were acquired. Each test was performed in triplicate. The tests were first conducted with 4 bar (gauge pressure) gas supplies for oxygen and air. The tests were then repeated with a portable oxygen concentrator (AirSep NewLife Intensity, CAIRE Inc.), together with a 1.3 bar (gauge pressure) air supply.

**Table 2:** Ventilator test conditions from ISO 80601-2-12:2020. I:E ratios were calculated from RR and inspiration time.

**Test no**.	***C* (ml/cmH_**2**_O)**	***R* [cmH_**2**_O/(l/s)]**	**V_**T**_ (ml)**	**RR (bpm)**	**Ins time (s)**	**FiO_**2**_ (%)**	**PEEP (cmH_**2**_O)**	**I:E**
Default	-	-	400	15	1	-	5	1:3
1	~50	~5	500	20	1	30	5	1:2
2		~20		12	1	90	10	1:4
3	20	5		20	1		5	1:2
4		20			1	30	10	
5			300		1		5	
6		50		12	1	90	10	1:4
7	10			20	1	30	10	1:2

The combination of test lung resistances and compliances for Tests 3–7 were implemented with the SmartLung. For Tests 1 and 2, the lung compliance of 50 ml/cmH_2_O was approximated by placing the EasyLung (R20/C25) in parallel with the SmartLung with R5/C30 or R20/C30, according to the resistance in Table 2. It should be noted that due to the different resistances on the two test lungs, the two compliances were not in parallel, and therefore not directly additive.

Flow and pressure measurements from the prototype were analysed from breaths 6 to 36, in order to ensure that the system had reached steady-state after input parameter changes. Tidal volume, PEEP, FiO_2_, and RR were calculated on a breath-by-breath basis, as previously described. The reference frames for these tests were: (i) V_T_ within 85%-4 ml to 115%+4 ml of target volume; (ii) achieved PEEP within ± 2 cmH_2_O of target value (ISO 80601-2-12:2020 allowable sensor error); (iii) average FiO_2_ within ± 5% of target oxygen concentration.

#### Durability Testing

Continuous ventilation was applied over a 15-day period to an R20/C25 test lung with the following clinical parameters: FiO_2_ of 21%, V_T_ of 400 ml, PEEP of 5 cmH_2_O, RR of 30 bpm, and an I:E ratio of 1:2. During the test, 30 s of data were acquired every 10 min by the prototype. The average inhaled volume and achieved PEEP over 15 breaths were calculated as previously described. The correlation coefficient between each parameter and time was used to evaluate any long-term changes in the ability of the prototype to deliver the required ventilation performance.

### Numerical Model

The dynamics of the ventilation achieved using on-off solenoids are dependent on their flow coefficients, and Design Principle 2 requires the ability to operate with components from different supply chains. In order to investigate the flexibility of the flow coefficients of Valves A-D, a numerical model of the system was built that could efficiently probe the performance of different valve combinations.

#### Model Design

A numerical model of the system was built in Matlab (The MathWorks, USA) using the Simulink design environment and physical components from the Simscape gas flow library. The lung was modelled as a parallel spring-and-dashpot circuit behind a translational motion interface that converted gas flow into displacement, similarly to the Simscape library model of a ventilator (21). This was utilised to convert the airway resistance to a damping coefficient and the reciprocal of the lung compliance to a stiffness. Neither gas exchange in the lung nor humidification of the exhaled air were accounted for in the model.

As Equations 1 and 5 capture the combined effects of both the valves and additional pneumatic components, the built-in valves, which function according to the ISO 6358 standard were augmented using a virtual pressure method (see Supplementary Figure S3 and corresponding text), so that the flow-pressure characteristics of the inhalation and exhalation pathway better mimicked those measured in the prototype.

#### Model Validation

To validate the numerical model, Bland-Altman tests were performed against the parametric prototype data. Airway resistance (*R*_*air*_) and lung compliance (*C*_*lung*_ = *V*_*T*_/(*p*_*plat*_ − *p*_*peep*_)) were estimated based on experimental data rather than the rated values for the reasons described in Section: Flow Analyser and Test Lungs. Tidal Volume, PiP, PEEP, and fraction of expiration time, $T_{ex}^{*}$, were averaged over 5 breaths. $T_{ex}^{*}$ represents the proportion of the exhalation time used to reach PEEP, defined as


(7) $$\begin{array}{c} T_{ex}^{*}=\frac{t_{6}-t_{3}}{T_{ex}} \end{array}$$

where *t*_*i*_ is the time at the beginning of Phase *i* (Figure 2) and *T*_*ex*_ = 60/*RR* × *E*/(1 + *E*).

#### Parametric Valve Evaluation

The main purpose of the numerical model is to identify ranges of valve flow coefficients that would enable appropriate ventilation characteristics (Design Principle 2). In order to relate the flow coefficients that would appear on valve specification sheets to the parameters in Equations 1 and 5, linear regression was performed between the flow coefficients and the power law constants for three different valves (Supplementary Figures S1C,D). The parameter *a* scaled linearly with flow coefficient, following *a* = 13.1 *K*_*v*_, while the power law exponent was insensitive to the valve flow coefficient.

Valve parameters were varied over appropriate ranges for commonly available components in order to identify usable flow coefficient ranges for each component. Deviating outside these ranges in either direction could have potentially deleterious consequences on ventilator performance. For Valves A and B, the flow coefficient determines how quickly *p*_*res*_ can increase and thus whether the Tidal Volume can be achieved. Delivered Tidal Volume is therefore used as an indicator of performance. For Valve C, a low flow coefficient (corresponding to a high pressure drop across the valve) would require a higher reservoir pressure to achieve Tidal Volume, and in the extreme, Tidal Volume would not be delivered. Conversely, a higher flow coefficient could also diminish performance as the lungs would be filled too quickly, resulting in higher inspiratory pressures. To determine usable Valve C flow coefficients, we therefore evaluated (i) the delivered Tidal Volume, (ii) PiP, and (iii) the plateau pressure (the pressure at the end of the breath), evaluated at the patient connector (*p*_*plat*_). Finally, the flow coefficient of Valve D determines the exhalation rate. If *K*_*v,D*_ was too low, PEEP might not be reached before the end of the breath and in extreme cases, breath stacking could occur. To evaluate Valve D, we evaluate $T_{ex}^{*}$, where $T_{ex}^{*}$ close to 1 would indicate a failure to achieve sufficiently rapid exhalation.

## Results

### Experimental Testing

**Figure 3:**
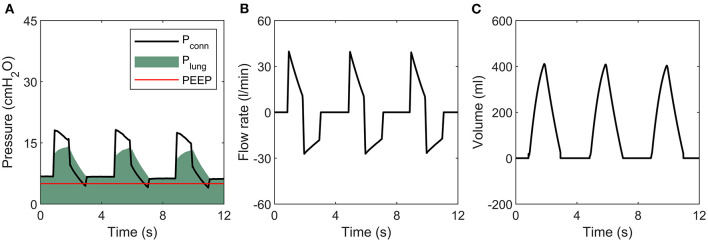
Sample tracing during ventilation of an R20/C20 lung. **(A)** Pressure, **(B)** Flow rate, **(C)** Volume.

The pressure, flow and volume traces (Figure 3) are repeatable and as would be expected to those familiar with ventilators, with one exception. In contrast to conventional systems, where expiratory flow decays exponentially towards zero, our system maintains high expiratory flow rates until Valve D closes when *p*_*lung*_ reaches PEEP. This is due to the novel exhalation pathway using an on-off type solenoid valve.

**Figure 4:**
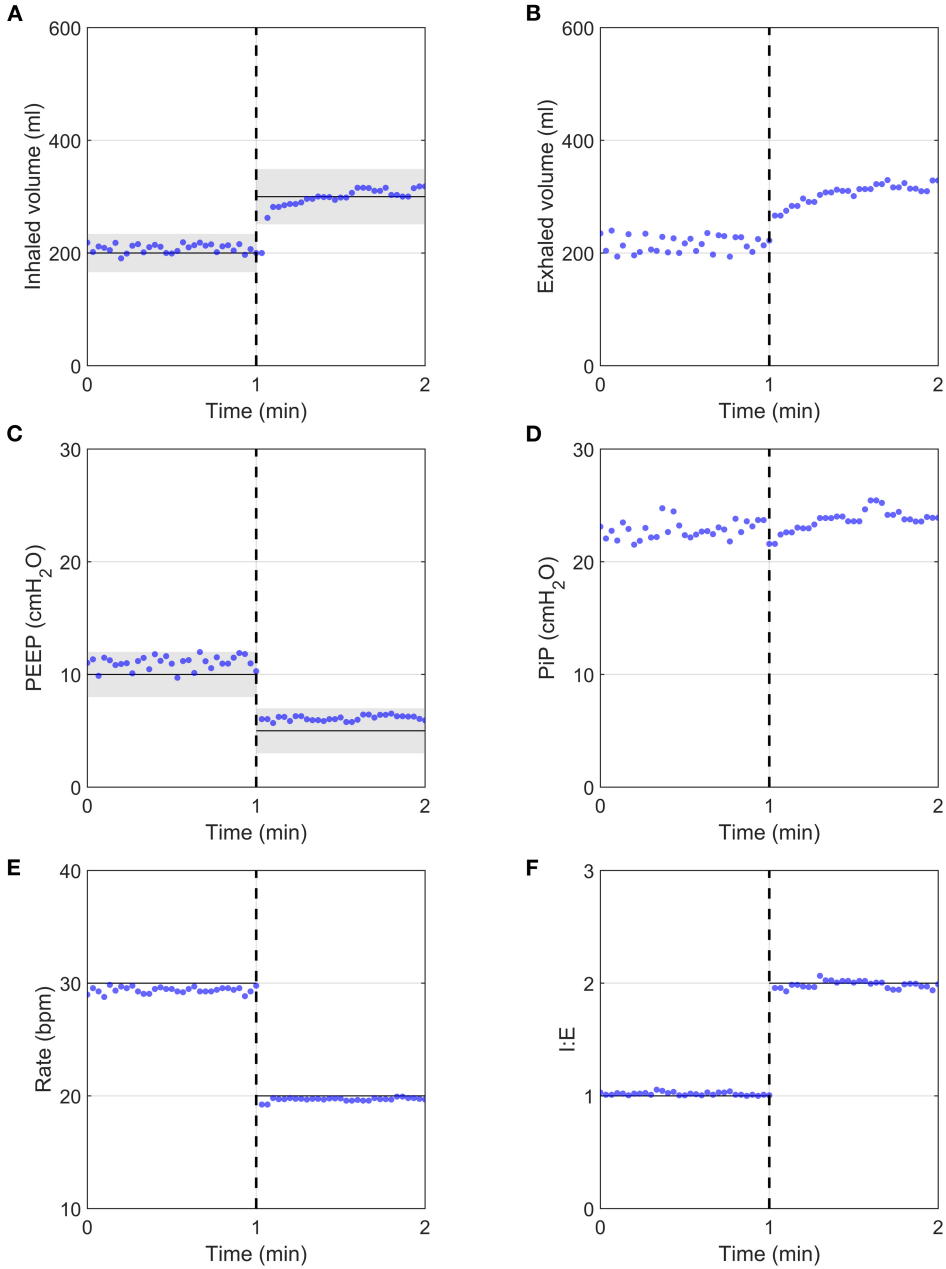
System response after the programmatic changing of all clinical parameters simultaneously (dashed vertical lines), measured using the flow analyser. Input parameters are indicated by continuous black horizontal lines, with ISO 80601-2-12:2020 measurement uncertainties indicated by grey shaded regions, with ± (4 ml + 15% of target value) for volume and ±2 cmH_2_O for PEEP. **(A)** Inhaled volume, **(B)** Exhaled volume, **(C)** PEEP, **(D)** PiP, **(E)** Respiratory Rate, **(F)** I:E ratio.

The prototype was able to respond rapidly to parameter changes within a few breaths, as demonstrated in Figure 4, in which PEEP, I:E ratio, RR, and V_T_ were changed simultaneously. PEEP and V_T_ values were within the acceptable margins of uncertainty set by ISO 80601-2-12:2020, delimited by the grey shaded region, immediately following parameter modification.

#### Measurement and Ventilation Accuracy

**Figure 5:**
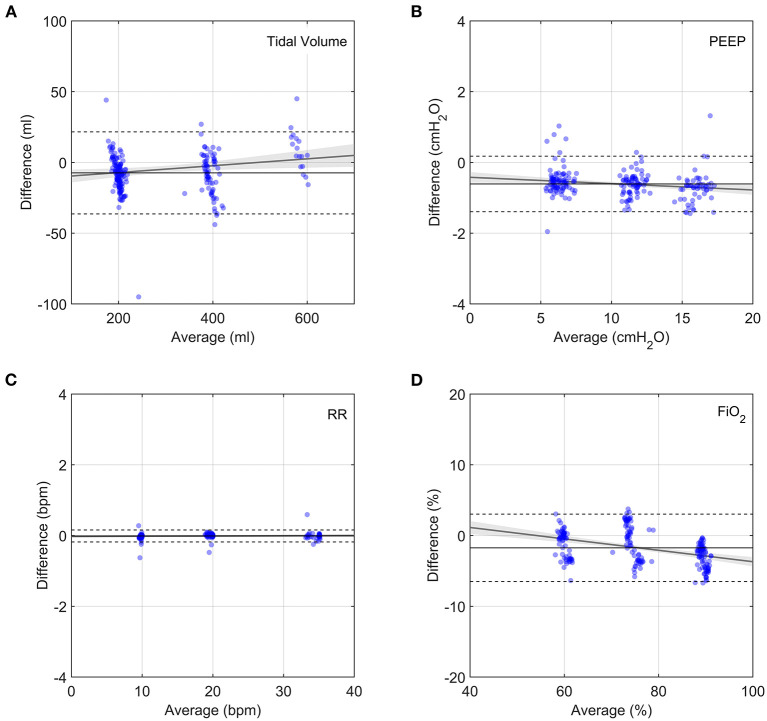
Bland-Altman plots of output parameters between the prototype and the flow analyser. Each data point represents the average of 9 breaths, taken 5 breaths after changing a single clinical input. Grey lines show linear fits to the data with the 95% confidence bands indicated by the shaded grey regions. Horizontal solid lines show average difference and dashed lines show plus and minus two standard deviations. **(A)** Tidal volume, **(B)** PEEP, **(C)** Respiratory Rate, **(D)** FiO_2_.

Bland-Altman analysis comparing the prototype to the flow analyser indicated acceptable measurement performance for all parameters (Figure 5 and Supplementary Table S2). Average differences were well within acceptable accuracy requirements. Although the average prototype output parameters were statistically different from those of the commercial flow analyser (Supplementary Table S2), the average differences were negligibly small. Low correlation coefficients (*R*^2^ < 0.2) indicate that parameter magnitude did not affect measurement accuracy. The prototype measurements can therefore be considered to be sufficiently accurate to determine system performance.

**Figure 6:**
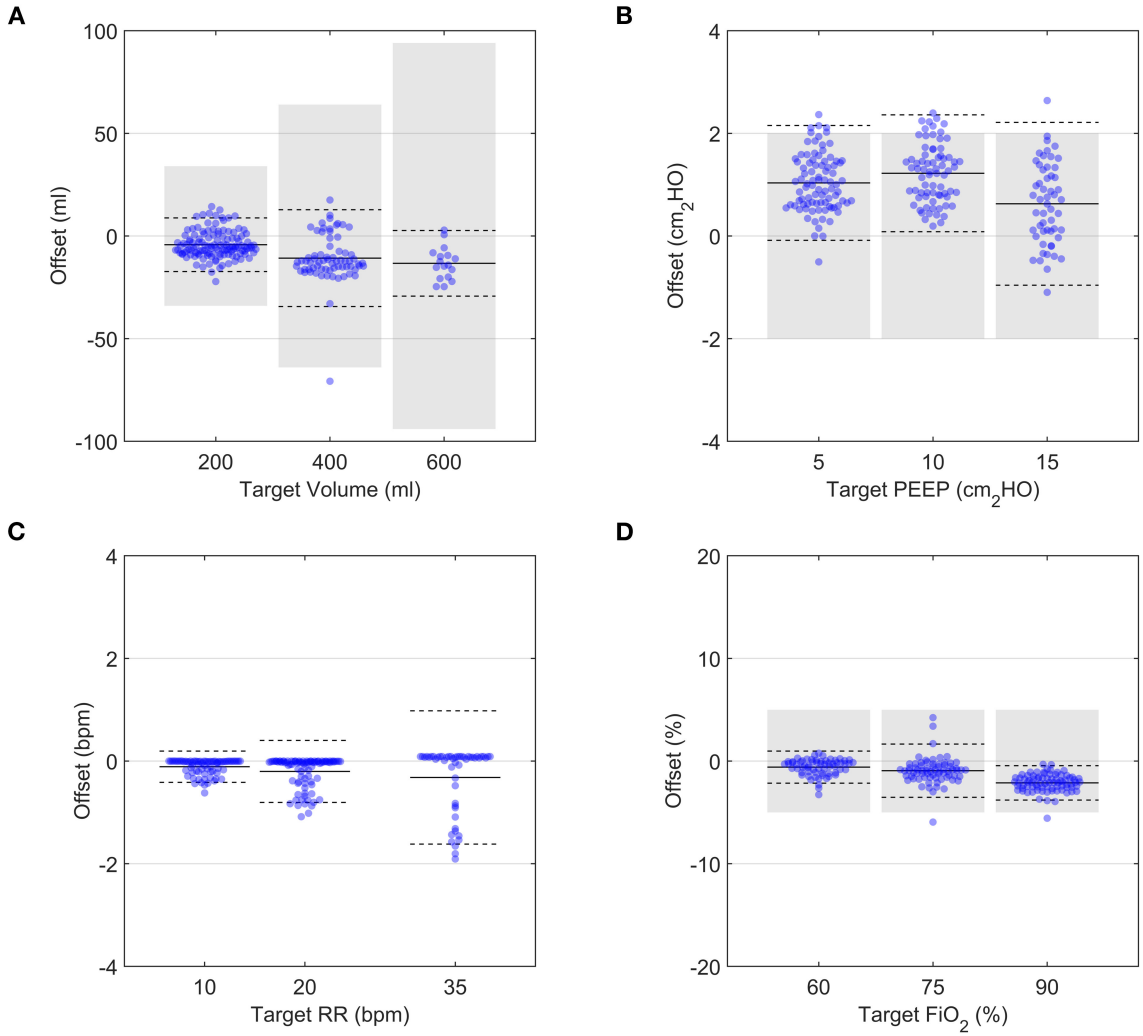
Demonstration of the performance over a range of setting permutations for an R20/C20 test lung configuration. Each data point represents the average of 9 breaths taken 5 breaths after a change in a single clinical input was implemented. In all panels, solid black lines and dashed black lines represent the average and 2 SD of all datapoints at a given target value, respectively. Grey shaded regions indicate ISO 80601-2-12:2020 measurement uncertainties. **(A)** Tidal volume, **(B)** PEEP, **(C)** Respiratory Rate, **(D)** FiO_2_.

For the parametric analysis, the prototype delivered the target ventilation parameters consistently (Figure 6) across the parametric range of ventilation conditions. Although the average offsets were significantly different from zero (Supplementary Table S3), both Tidal Volume and FiO_2_ targets were achieved within acceptable uncertainty standards (±15% of *V*_*T*_ and ±5% FiO_2_, respectively). PEEP was also consistently within the pressure accuracy requirements of ±2 cmH_2_O, with a tendency to deliver slightly higher values. This was due the control algorithm being optimised to never go below PEEP, as required by the MHRA RMVS guidelines. Respiratory rate was accurate, with a few outlying cases presenting a small lag. Hence, the system performed well over a whole range of ventilation parameters, with only small offsets from target values.

#### ISO 80601-2-12 Testing

**Figure 7:**
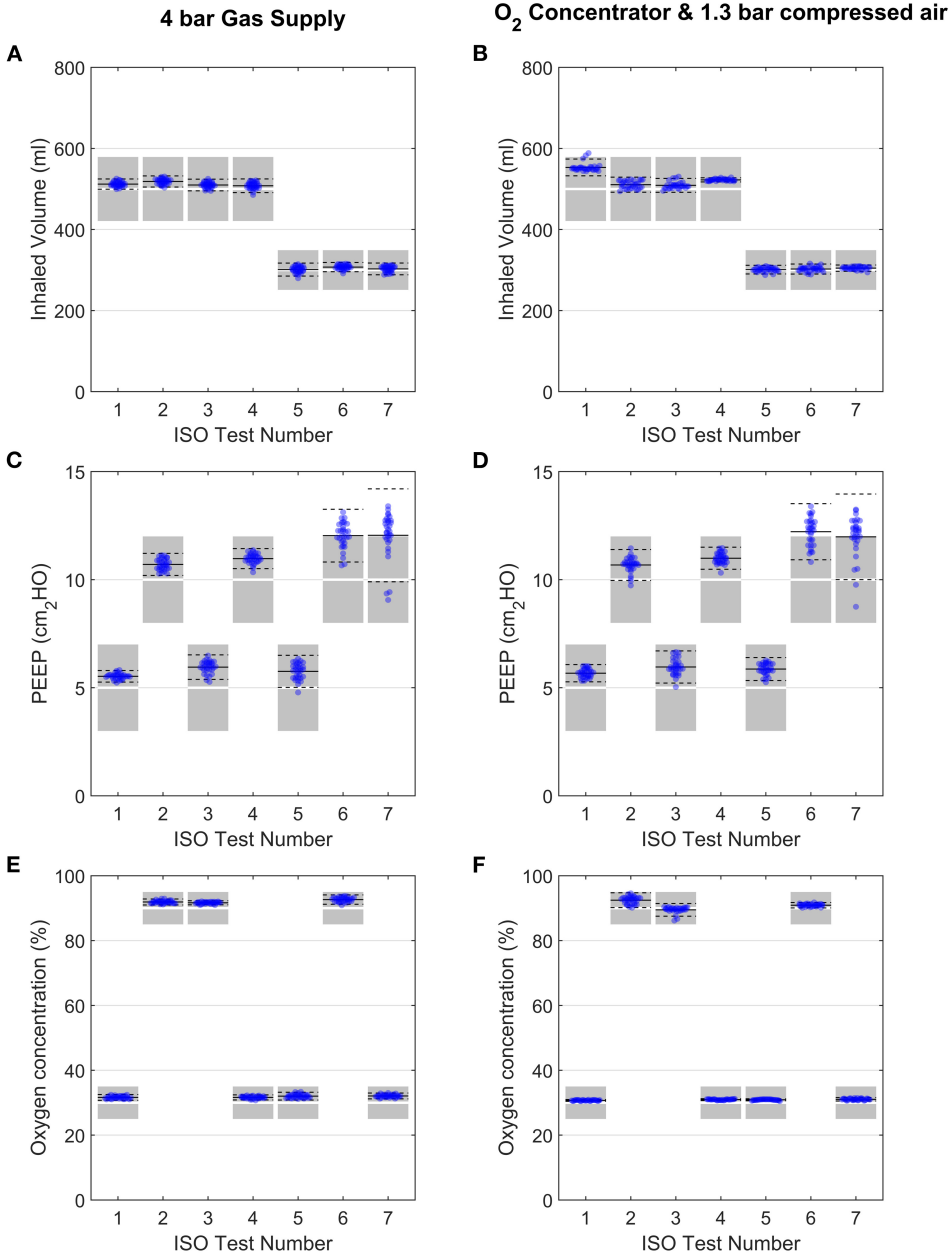
Evaluation of performance under the ISO 80601-2-12:2020 testing schedule (Table 2). Variation in inhaled volume, PEEP and oxygen concentration are shown for each ISO test, when used with 4-bar gas supplies **(A–C)** or an oxygen concentrator **(D–F)**. Each data point represents for 1 breath, with 31 breaths shown for each test. Data points shown are from breaths 4 to 36 (exclusive). In all panels, solid black lines and dashed black lines represent the average and ± 2SD of all datapoints. Shaded regions in panels **(A,B,E,F)** represent ISO 80601-2-12:2020 acceptable margins of uncertainty [± (4 ml + 15% of the measured value) for volume and ±5% for oxygen concentration], while those in panel **(C)** and **(D)** represent the ISO 80601-2-12:2020 acceptable uncertainty in PEEP pressure measurement (±2 cmH_2_O).

Using 4-bar pressure supplies (mimicking typical hospital gas lines), the system performance was consistent for the range of lung configurations set out by ISO 80601-2-12:2020 (Figures 7A,C,E, see Supplementary Figures S4–S10 for representative traces of each case). Oxygen concentration and inhaled volume were highly repeatable for all 7 tests (see Supplementary Table S4). The PEEP control performed marginally less well for Cases 6 and 7 (both R50), with average values 12.0 ± 1.2 and 12.1 ± 2.1 cmH_2_O with the 4 bar gas supply (target value was 10 cmH_2_O). However, these deviations are small, and exhalations were completed well within the exhalation period.

The same tests were then carried out with a home-use oxygen concentrator and 1.3 bar air supply, with no changes made to the control algorithm settings (Figures 7B,D,F and Supplementary Table S4). The results demonstrated no negative effects on the performance, indicating the system is robust to a range of gas supply options.

#### Durability Testing

**Figure 8:**
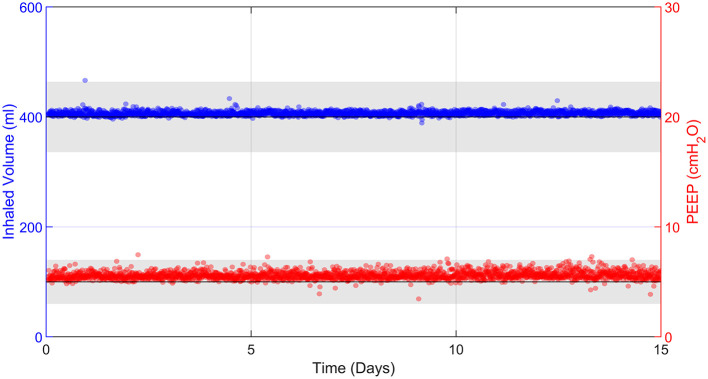
15 days of uninterrupted ventilation using the prototype system and R20/C25 test lung. Each data point represents 30 s of averaged data every 10 min. Grey bands represent the ISO 80601-2-12:2020 acceptable uncertainty in Tidal Volume, ± (4 ml + 15% of the measured value), and the ISO 80601-2-12:2020 acceptable error in PEEP pressure measurements, ± 2 cmH_2_O.

In order to comply with Design Principle 4, the prototype must be able to function robustly over long time periods with no operator intervention. Over 15 days, the Tidal Volume was stable with an average of 407 ± 8 ml (mean ± 2 SD) (see Supplementary Table S5). The correlation coefficient between time and Tidal Volume was *R*^2^ = 0.01, indicating no relevant change over time. Similarly, the average PEEP was 5.6 ± 0.7 cmH_2_O (Figure 8), which is within the allowable accuracy ranges, with *R*^2^ = 0.02. This demonstrates the robustness of the prototype over clinically relevant ICU timescales.

### Numerical Model Testing

#### Model Validation

**Figure 9:**
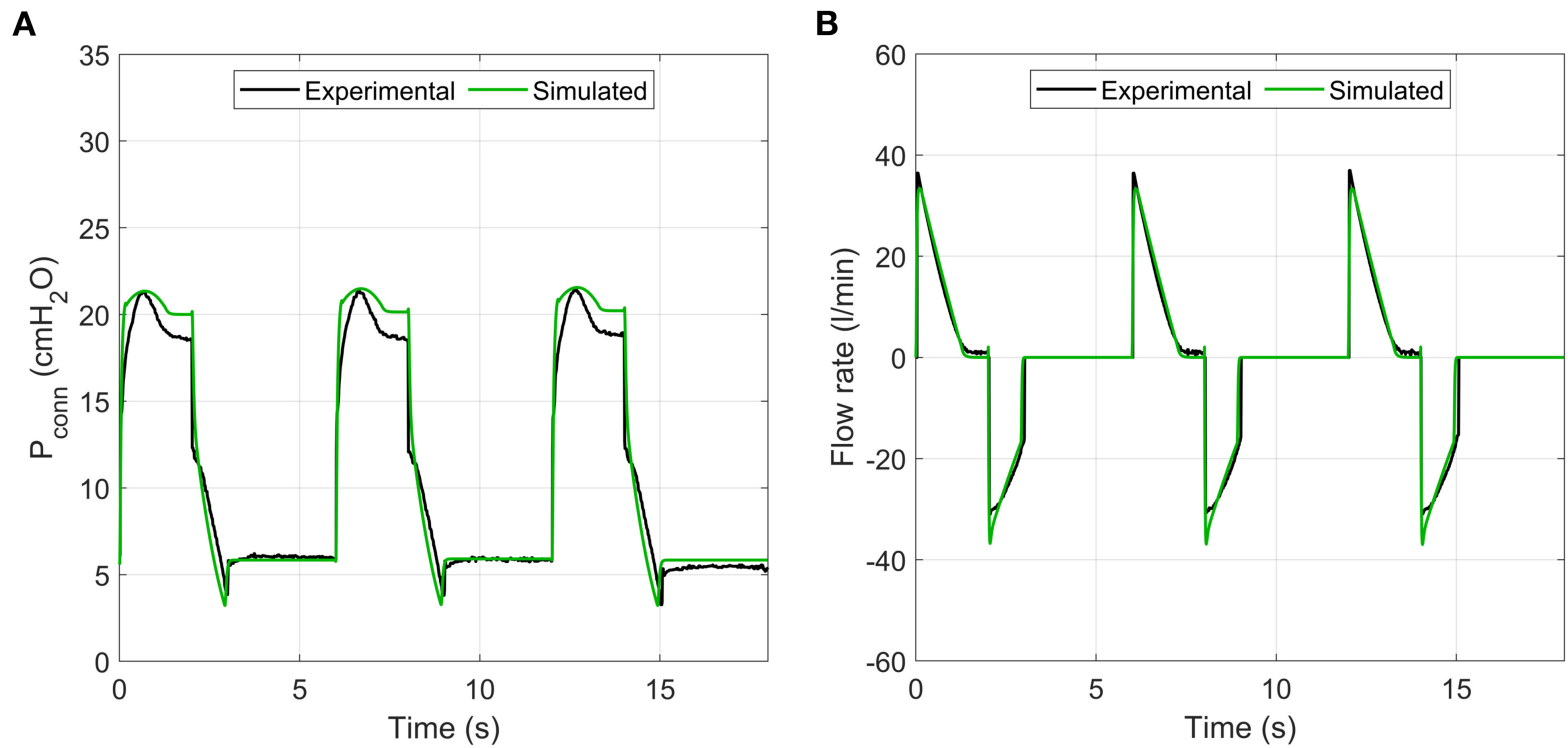
Sample comparison of pressure and flow rate traces between experimental prototype and numerical model. **(A)** pressure, **(B)** flow rate.

**Figure 10:**
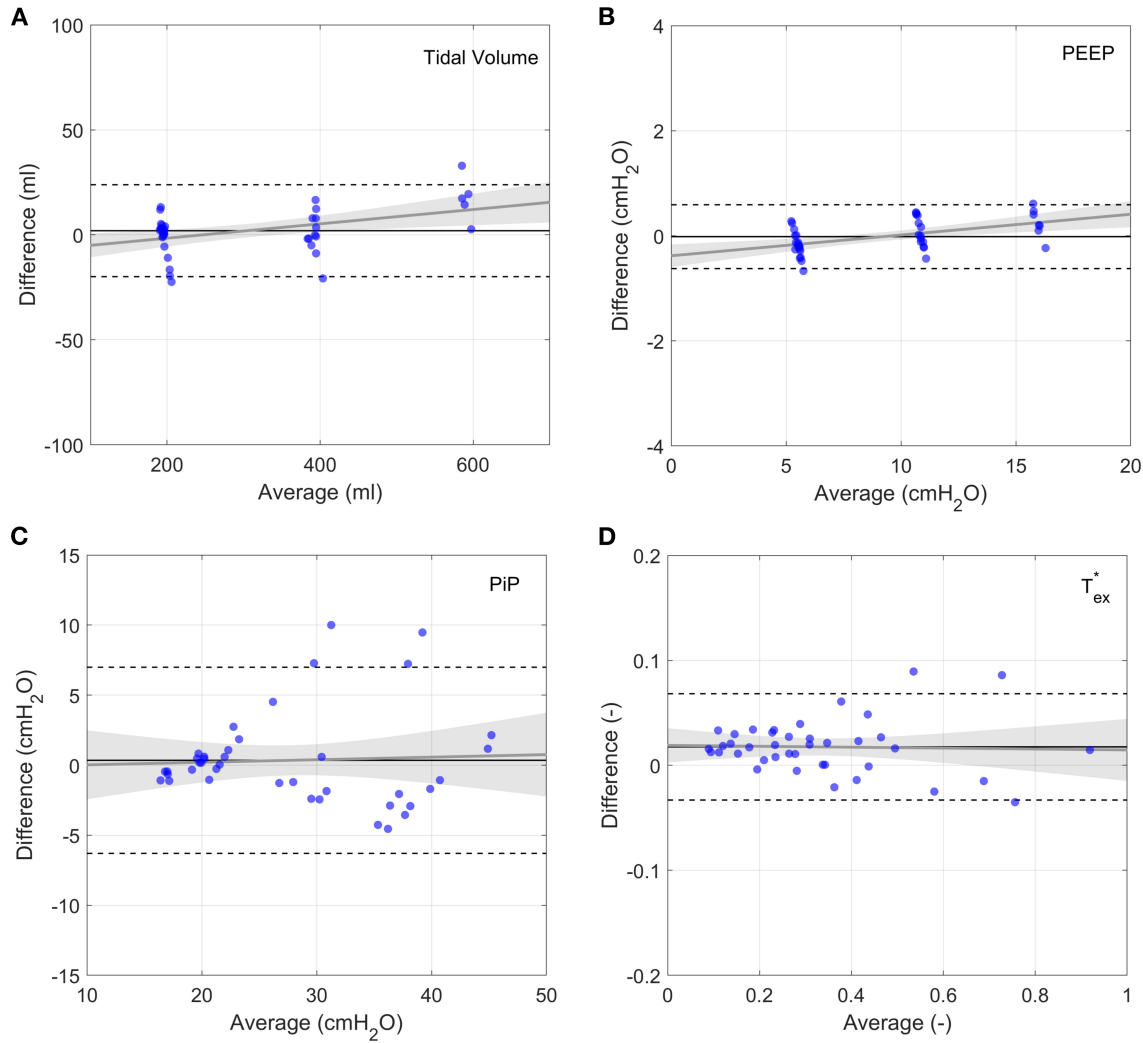
Bland-Altman plots of output parameters between the numerical model and experimental data. Each experimental data point represents the average of 9 breaths, taken 5 breaths after changing a single clinical input. Grey lines show linear fits to the data with the 95% confidence bands indicated by the shaded grey regions. Horizontal solid lines show average difference and dashed lines show plus and minus two standard deviations. **(A)** Tidal volume, **(B)** PEEP, **(C)** PiP, **(D)** ${\text{T}}_{\text{ex}}^{*}$.

The numerical model reproduced the key features of the pressure and flow waveforms delivered to the patient (Figure 9). Minor differences, particularly around inspiration pressure, were due to the idealised representations of the mock lungs used in the numerical model. Direct comparison with Bland-Altman analysis showed consistent agreement with the typical clinical outputs Tidal Volume, PiP and PEEP (Figure 10 and Supplementary Table S6) under the variety of conditions tested in the benchtop parametric sweep. The model-to-experimental differences of Tidal Volume correlated positively with the average values (*p* < 0.01), but the deviations were within 10% of the average Tidal Volume (Figure 10A). A similar result was observed for PEEP, with a small (<1 cmH_2_O) dependence of the difference on the average (*p* < 0.001, Figure 10B). There was no systematic bias for PiP (*p* > 0.7) but the difference between model and prototype increased with the average value (Figure 10C) due to differences between the idealised model lungs and the test lungs used for the experiments. No systematic error in the parameter $T_{ex}^{*}$ was observed (*p* > 0.8) with differences of <10% between the experiments and model.

#### Numerical Model Predictions of Robustness of Ventilation to Valve Flow Coefficients

System performance was robust to the flow coefficient of the air and oxygen valves (A and B). The Tidal Volume was achieved for all $K_{v,A/B}\geq 0.02\;m^{3}/h/bar^{0.5}$ with 4 bar gauge pressure supply (Supplementary Figure S11A). For 1.3 bar gauge supply pressure, as provided by an oxygen concentrator, Tidal Volume was achieved for $K_{v,A/B}>0.04\;m^{3}/h/bar^{0.5}\;$(Supplementary Figure S11B).

**Figure 11:**
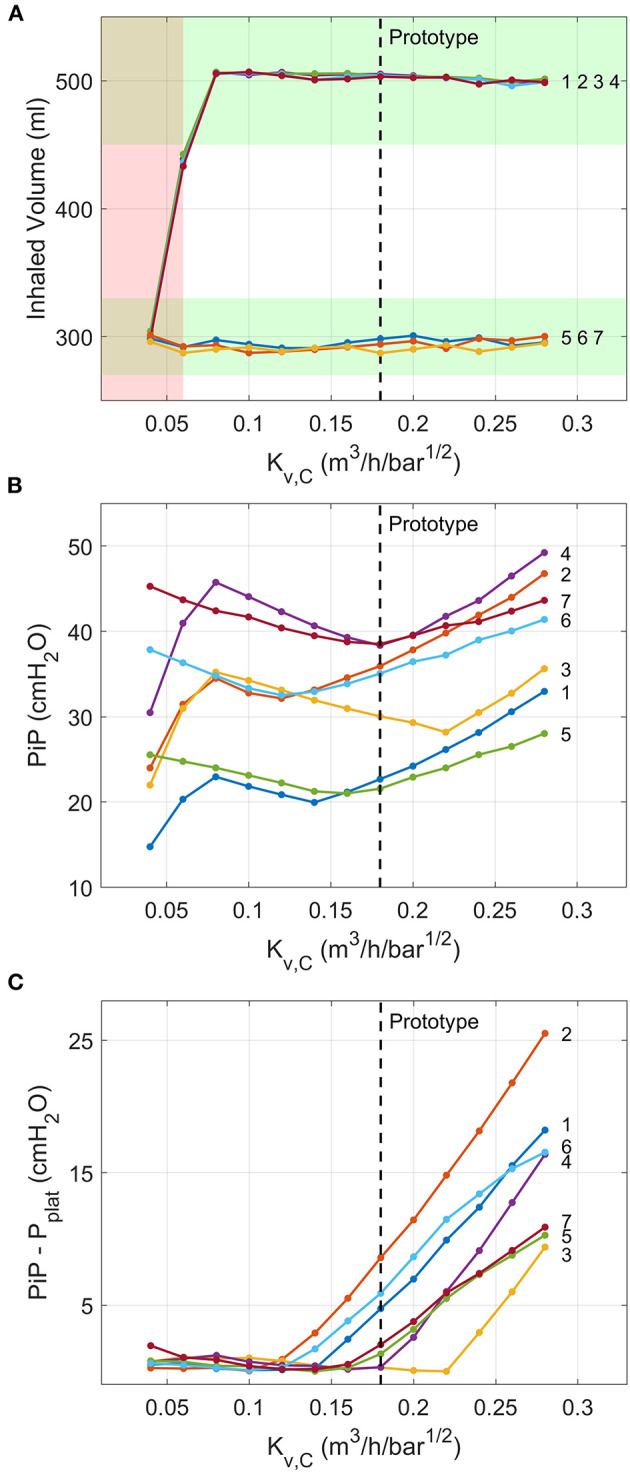
Numerical model predictions of the variations in critical outputs of the inspiration pathway with the flow coefficient of Valve C. Ventilation conditions are labelled by their numbers according to the ISO 80601-2-12:2020 test requirements (Table 2). **(A)** Tidal volume, with the ISO requirement of being within 10% of the target values shaded green and ranges of *K*_*v,C*_ that fail to meet ISO specifications shaded red. **(B)** PiP, and **(C)** difference between PiP and *p*_*plat*_. Vertical dashed lines indicate flow coefficient of valve used in the prototype.

**Figure 12:**
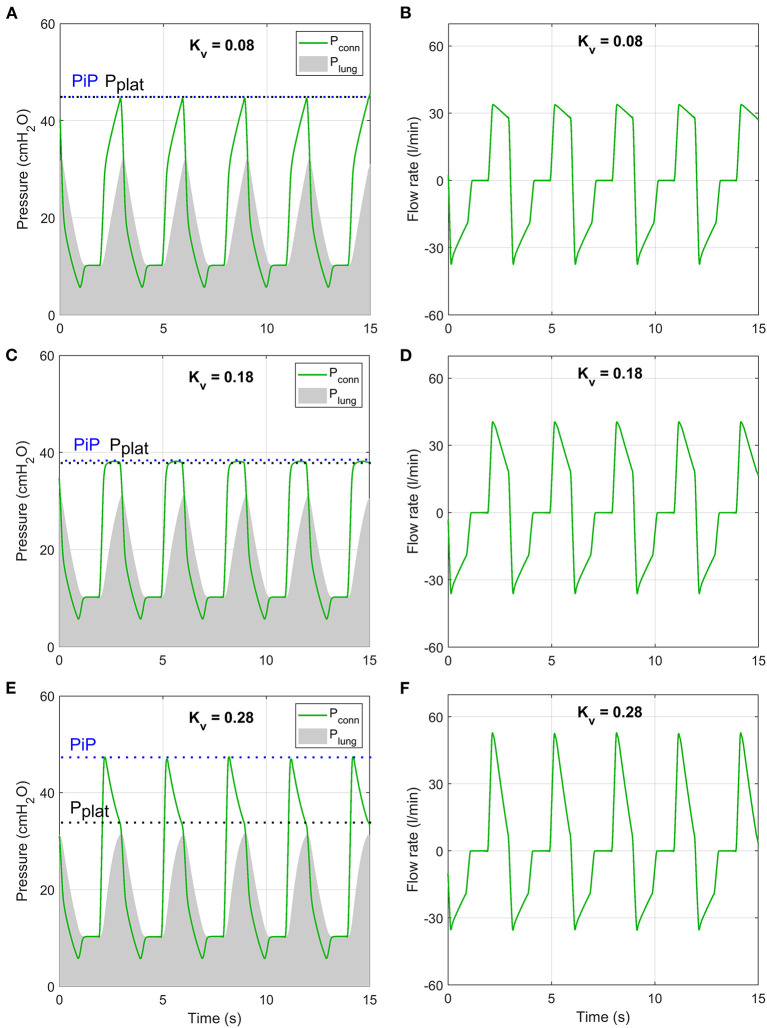
Numerical model predictions of pressure **(A,C,E)** and flow rate **(B,D,F)** under conditions of ISO 80601-2-12:2020 test case 4 (purple lines in Figure 11) at three values of *K*_*v,C*_ that are representative of three inhalation regimes.

According to the inhalation valve analysis, Tidal Volume was achieved for all ISO 80601 test cases for $K_{v,C}>0.06\;m^{3}/h/bar^{0.5}$. For lower *K*_*v,C*_ values, Tidal Volumes of 500 ml were not achieved (Figure 11A). Peak inspiratory pressures varied non-monotonically as *K*_*v,C*_ changed (Figure 11B). The cause of this becomes clear when considering sample traces, as shown in Figure 12 for ISO Test 4. There are three regimes that occur for different *K*_*v,C*_ values and ventilation parameters. The low-*K*_*v,C*_ regime is characterised by a slowly increasing airway pressure and relatively constant flow rate (Figures 12A,D), similar to a VCV ventilation mode. In the medium-*K*_*v,C*_ regime, the airway pressure reaches its plateau rapidly, approximating a PCV system (Figure 12B). The flow rate has a higher initial value and decreases by ~60% by the beginning of expiration (Figure 12D). The value used in the prototype ($K_{v,C}=0.17\;m^{3}/h/bar^{0.5}$) fits within this regime. The high-*K*_*v,C*_ regime is characterised by a high initial peak airway pressure that drops down to a plateau pressure at the end of inspiration (Figure 12E). The high PiP makes this profile less desirable. However, it should be noted that for all three regimes, the peak lung pressure is approximately the same, so the high peak pressures at the connector may not be critical for patient comfort.

**Figure 13:**
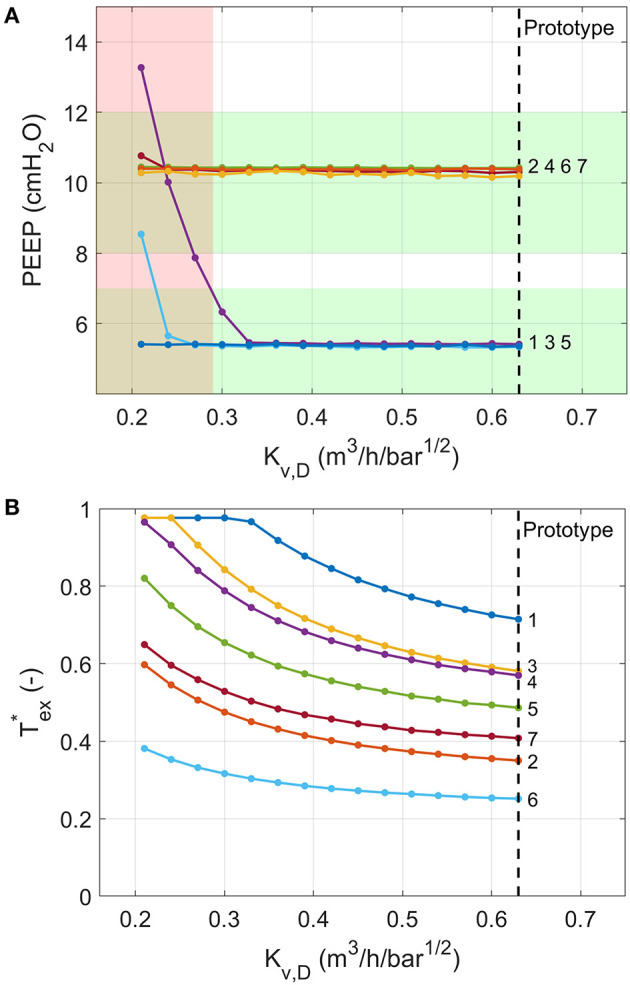
Numerical model predictions of the variation in **(A)** PEEP and **(B)** the fraction of exhalation time required to reach PEEP for different values of the flow coefficient of Valve D. Ventilation conditions are labelled by their numbers according to the ISO 80601-2-12:2020 test requirements (Table 2). The red shading indicates a failure in at least one of the test cases (PEEP more than 2 cmH_2_O away from target), predicted for *K*_*v,D*_ ≤ 0.29. Vertical dashed lines indicate flow coefficient of valve used in the prototype.

PEEP was achieved for all ISO 80601 cases with $K_{v,D}>0.29\;m^{3}/h/bar^{0.5}$ (Figure 13A). Below this value, expirations terminate at higher pressures because the pressure-decay time (characterised by $T_{ex}^{*}$) is insufficient (Figure 13B). In general, higher values of *K*_*v,D*_ reduce $T_{ex}^{*}$ and hence reduce the chance of breath stacking in extreme cases of high lung resistance or high respiratory rates.

## Discussion

In this study, we described a novel ventilator design, intended to address the global shortage of ventilators that was exacerbated by the COVID-19 pandemic. We established a series of design principles to guide our development process towards a technology that would be viable for long-term ICU usage, whilst minimising dependence on specific supply chains. The core feature of our solution is the usage of solely on-off solenoid valves in place of the proportional solenoid valves or mass-flow controllers that are typically used in ICU ventilators. In providing only two states of control, on-off valves are less flexible than their proportional counterparts, which may be a reason why this approach has not commonly been used for ventilator design. However, this simplicity also makes the control process more straightforward, requiring only appropriate timing control to achieve the required ventilation performance. We demonstrated, *via* rigorous evaluation with commercially available test lungs and a gas flow analyser, that our prototype can provide the ventilation performance required by emergency-use approvals (e.g., MHRA RMVS) and ISO regulatory approvals.

### Advantages of a Design Based on On-Off Valves

The use of on-off valves provides several clear advantages. Firstly, such components are available from numerous suppliers across the world. As we demonstrated with our numerical model (Figures 11, 13 and Supplementary S11), the system performance is robust to ranges of valve flow coefficients without any changes required to the control algorithm. As such, it should be possible to build multiple “realisations” of our design for different supply chains. The ranges of available valve flow coefficients, *K*_*v*_, vary between manufacturer and series and are largely dependent on the orifice size. For example, the ASCO 262 series valves used in the prototype provide *K*_*v*_ values from 0.05 up to 0.76 m^3^/h/bar^1/2^. Smaller form-factor valves, such as the ASCO RB series and IMI Microsol series, provide lower *K*_*v*_ values, of 0.02–0.10 m^3^/h/bar^1/2^ and 0.009–0.18 m^3^/h/bar^1/2^, respectively. The *K*_*v*_ values required for Valves A-C are therefore common. The higher *K*_*v*_ values required for Valve D are less easily sourced, as they require *K*_*v*_ > 0.3 m^3^/h/bar^1/2^. However, if valves with higher *K*_*v*_ were not available, then the effective *K*_*v*_ could be approximately doubled by placing two valves of similar orifice size in parallel.

Secondly, as on-off type valves have a single, characterisable flow-pressure relationship (Supplementary Figure S1), there was no need for flow meters (typically used in both inhalation and exhalation pathways), which reduced the number of components and thus cost. In comparison, proportional valves cannot be used in this manner, due to hysteresis and different flow pressure relationships at every valve position.

Thirdly, the simplicity of on-off valves means that they are cheap to purchase and easy to maintain or repair when seals wear out. For manifold-based valves (such as IMI FAS Microsol series or ASCO RB series) replacement only involves a few screws for complete replacement and can thus be carried out in minutes. Depending on the supplier and series, the rated lifetime of these valves varies between 10 and 100 million cycles. Assuming 20 breaths per minute, this equates to ~1–10 years of continuous ventilation. Designs that use solenoid valves as the only moving parts also offer clear advantages over systems that have large moving parts, such as designs including bellows typically used for anaesthetic ventilators (Design Principle 3).

Fourthly, the configuration of valves means that there are two levels of isolation of the patient from the gas supply on the inhalation pathway. These valves are “normally closed,” meaning that in the event of a power failure the patient would still be isolated from high pressure gas. Additionally, the exhalation valve is normally open, so that if there was a power supply failure, the patient would be able to breathe atmospheric air. The addition of two levels of pressure relief valves makes the design inherently safe.

Finally, the system has low power requirements. On-off valves have a straightforward electronic function, operating at 12 or 24 VDC and only requiring a full current draw to activate, after which a lower current suffices to maintain the active state. This provides an advantage over proportional solenoid valves that require continuous current input for controlling the orifice and have rated powers that are typically greater than or equal to that of on-off valves for a given orifice size. The current prototype uses the larger 262 series valves that require ~10 W to hold in the active state, but as there are only one or two valves open at a time, the actual power usage is likely <20 W. Smaller form factor valves, such as the IMI FAS Microsol series or ASCO 096 series, require only 2 W to hold in the active state, so power usage could be significantly reduced with these valves. The prototype can be run from a medical PC that typically uses ~40–50 W at 24 VDC, but future versions could use simple microprocessors and touch screens to further reduce power consumption. All sensing components can also be run from a DC voltage supply, making the design compatible with DC battery power as a lightweight backup that could keep the ventilator running without mains power. This is preferable in terms of weight and electrical safety to using an Uninterrupted Power Supply, which provides high voltage AC power in the absence of a mains connection.

Using the numerical model, we were able to reproduce the behaviour of the system captured during the experiments. The model provides a tool that can be used to evaluate valve flow coefficients for different supply chains, in line with Design Principle 2. The model could also be used to evaluate extensions of the design, such as compatibility with paediatric performance requirements or incorporation of air entrainment for ambulatory applications.

### Disadvantages of a Design Based on On-Off Valves

Despite the above advantages, the main disadvantage of on-off valves is the restrictions that they put on the flexibility of the ventilator to additional modes of ventilation. We have demonstrated in the present study that the clinically preferred PRVC mode can be achieved with these components, and additional testing (not shown) has demonstrated that the system can operate in pressure control mode and triggered breathing modes. However, with simplicity inevitably comes a reduction in flexibility. Proportional valves allow precise regulation of flow rate or pressure, for advanced ventilation modes, such as APRV (airway pressure release ventilation), although it remains unclear whether these modes really offer clinical benefit (22, 23). We also cannot provide an automated control of flow rate for NIV, which is found in many ICU ventilators, but manual control could easily be incorporated if required.

The other minor disadvantage of the use of on-off valves, and specifically the use of the pressure drop across the valve to measure flow rates, is that the parameters in Equations 1 and 5 require calibration. Initial calibration would be carried out in the factory, but as with all ventilators, regular calibration would be required as part of general maintenance, so it is not expected that the calibration burden would be any different in this context. Long-term testing would be required to evaluate how much the parameters *a* and *n* vary between individual systems and for the same system over time.

### System Performance

We used commercially available test lungs to evaluate the performance of our prototype, which achieved excellent performance across the range of parametric tests as well as those set out in ISO 80601-2-12:2020. We also demonstrated consistent, accurate operation over 15 days, which is critical for long term use. Furthermore, with no changes to the control algorithm, the performance on the ISO tests was not affected when using a home-use oxygen concentrator that generated 1.3 bar rather than a compressed oxygen supply at 4 bar, making the design flexible for deployment to environments where hospital gas supply lines are unavailable and cylinders are in short supply.

In 2014 L'Her et al. reviewed the Tidal Volume and PEEP performance characteristics for 26 different ventilators (classified as ICU-like, Sophisticated, Simple, and Mass casualty response ventilators) using a range of lung resistances and compliances (24). While there are variations between the ventilator and lung settings used, our design achieved performance consistent with the ICU-like and Sophisticated ventilators described therein.

Programming the system in LabVIEW enabled its rapid development but limited its response time to ~20 ms, whereas hardware with lower-level programming would be able to achieve 1 ms response times without difficulty and will be implemented in future prototypes. With a faster response time, the accuracy in PEEP, particularly for ISO cases 6 and 7, would be improved. The offset in PEEP of consistently ~1 cmH_2_O arose from the MHRA RMVS requirement for PEEP never to go below the target value, although ISO regulations do not specify this.

### Supply Chain Factors

One of the key motivators behind the design, as per Principles 1 and 2, was to keep the supply chain overlap between our new design and existing regulatory-approved designs to a minimum. In addition to control valves, there are other core components in the system that should be considered, such as oxygen sensors and electronics.

Valves: as discussed, a major bottle neck in the supply chain was mass flow controllers, proportional valves and flow meters. We addressed this through the use of on-off solenoid valves.

Oxygen sensors: the oxygen sensors used in ventilators are typically electro-galvanic fuel cells that utilise the reaction between lead and oxygen to produce a current. There are unofficial reports of shortages of these components during the pandemic. Monitoring the amount of oxygen delivered to the patient (FiO_2_) is an important safety feature to avoid hyperoxia or hypoxia. Our design incorporates an oxygen sensor to increase the accuracy and response rate of the control algorithm for regulating oxygen concentration. However, due to the use of on-off solenoid valves, the delivered oxygen concentration at steady state is directly related to the proportion of time that the Oxygen and Air Inlet Valves are open, provided the supply pressures are equal. Hence, it would be straightforward to build a version of our design without an oxygen sensor that would provide similar accuracy, albeit with a longer response time to a change in FiO_2_.

Pressure Sensors: it is vital that pressure is monitored during ventilation in order to avoid barotrauma and achieve accurate PEEP control. Our design uses two pressure sensors, which is typical. Indeed, many ICU ventilators typically require more for accurate operation of their control algorithms.

Electronics: to achieve ICU-level ventilation performance, all modern ventilators require some level of electronic componentry. The circuitry required to drive on-off valves (a relay with software to minimise power usage in the active position) is much simpler than the current regulation circuits typically required to control proportional valves (for which inaccuracy in current will directly affect pressure). Additionally, as the control algorithm predominantly requires only evaluation of timing control (Figure 2), it can be easily re-written for different microcontrollers. Indeed, we have a functional prototype PCB version of our design based on an Teensy Microcontroller (PJRC).

In summary, while our design is still dependent on the supply chain, it requires fewer specialist core components and offers several advantages as a result of the simplicity of the design in line with Design Principles 1 and 2.

### Lung Models

A limitation of this study is the lack of human or animal data to validate the system. However, healthy animal lungs would not be able to realistically replicate the high-resistance, low-compliance state of pathological human lungs in respiratory disease, so such data would not significantly enhance the current analysis. Human trial data is essential for evaluating the ability of the proposed design to deliver efficient and comfortable ventilation to patients, and it will be essential to demonstrate equivalence with existing ventilators to achieve full regulatory approval. However, our benchtop testing evaluation has demonstrated that the proposed design is capable of delivering the required performance over a range of clinical parameters and model lung configurations. The only notable difference between the current approach and standard ventilators is the shape of the flow curve during exhalation (Figure 3) where the flow rate rapidly drops to zero at PEEP, rather than following an exponential decay. However, as the pressure falls steadily to PEEP, we do not expect this to result in any discomfort for the patient. Furthermore, this response offers the significant benefit of rapid exhalations for challenging lung dynamics, (particularly high resistance and high compliance) which reduces the risk of breath stacking.

While the numerical model was successfully validated against the *in vitro* experimental data in test lungs, the lumped parameter representation of the lungs (used in both the experiments and numerical model) has limitations. The series resistance and compliance model, which is well-accepted for the purposes of preclinical testing and forms the basis of the relevant ISO standards, is a significant simplification. Furthermore, the model assumed constant resistance and compliance, which was not the case for the experimental test lungs (Supplementary Figure S2). The adult human airway network is a combination of millions of dynamic resistors and non-linear compliance elements in series and parallel. Several authors have proposed improvements to these models, such as including a series inertance to represent the upper airways in combination with a constant-phase complex impedance to represent the alveoli (25). This approach has been shown to reproduce lung behaviours for a wide range of conditions, including asthma (26) or acute lung injury (27). Others have proposed including a non-linear lung elastance (28), or accounting for spatially varying degrees of alveolar unit recruitment, which are common in disease (29). Such adaptations to the model are outside the scope of the current study but could be useful in refining control algorithms following clinical trials.

### Future Development

In order to convert the prototype to a deployable medical device, the emergency design will need to be re-developed under an ISO13485 framework. This study demonstrates that the concept is sound. Additionally, in this study we demonstrated a pressure-regulated volume control mode, but the prototype can also deliver pressure regulated and spontaneous (triggered) breathing modes. The latter allows supported breaths triggered by patient effort that enables weaning from ventilation, a critical part of the recovery process. This additional functionality is currently undergoing rigorous evaluation with custom testing equipment designed to simulate patient effort.

### Conclusions

We demonstrated that our prototype is accurate, durable and flexible to different gas supply pressures. Together with low power usage and robustness, this makes the design ideal for use in emergency settings for COVID-19. Furthermore, these same characteristics make the system ideal for use in low and middle income countries and newly emerging economies, where respiratory conditions such as tuberculosis, malaria and influenza result in millions of deaths every year (30, 31). Resolving the huge inequity in access to life-saving medical devices is a complex problem, requiring not only the ventilators, but the infrastructure around them, including power, compressed gas supplies, hospital beds, suitably trained clinical staff and available engineering skills for repair and maintenance. While a ventilator design cannot address all of these factors, through simplicity of manufacture and operation, we believe that this approach has potential to contribute to this important mission.

## Data Availability Statement

Data from the ISO tests (section ISO 80601-2-12 Testing and Supplementary Figures S4–S10) is available from 667 https://doi.org/10.6084/m9.figshare.14562201.

## Author Contributions

MM, WB, JF, DW, AM, MS, JM, JM-L, and JvB-S contributed to conception and design of the study and wrote sections of the manuscript. JvB-S, MM, and JM-L developed and built the prototype hardware. JvB-S, MM, and AM developed and built the prototype software. MM, DW, and JF acquired and analysed experimental data. WB built the numerical model and analysed the data. All authors contributed to manuscript revision, read, and approved the submitted version.

## Funding

This project was supported by the Royal Academy of Engineering under the Research Fellowship Scheme (RF201617\16\18: JvB-S) and Pandemic X Preparedness Scheme (EXPP2021\1\317), and the Imperial College Presidents' COVID relief fund. JM acknowledges the support of the Sir Leon Bagrit Trust.

## Conflict of Interest

MM, JM-L, and JvB-S are named inventors on a patent application for the exhalation pathway of this ventilator design. MM, WB, JF, DW, JM, JM-L, and JvB-S are founders of the spinout company Phaedrus World Medical that aims to commercialise this ventilator. The remaining authors declare that the research was conducted in the absence of any commercial or financial relationships that could be construed as a potential conflict of interest.

## Publisher's Note

All claims expressed in this article are solely those of the authors and do not necessarily represent those of their affiliated organizations, or those of the publisher, the editors and the reviewers. Any product that may be evaluated in this article, or claim that may be made by its manufacturer, is not guaranteed or endorsed by the publisher.
