# Supplementary material for: A Novel Ventilator Design for COVID-19 and Resource-Limited Settings
Source: Front Med Technol. 2021 Oct 4;3:707826. doi: 10.3389/fmedt.2021.707826 (PMC8757831; doi:10.3389/fmedt.2021.707826)
Supplement: Supplementary file 1 [file Data_Sheet_1.pdf]

Supplementary Material

Flow Rate Calibration

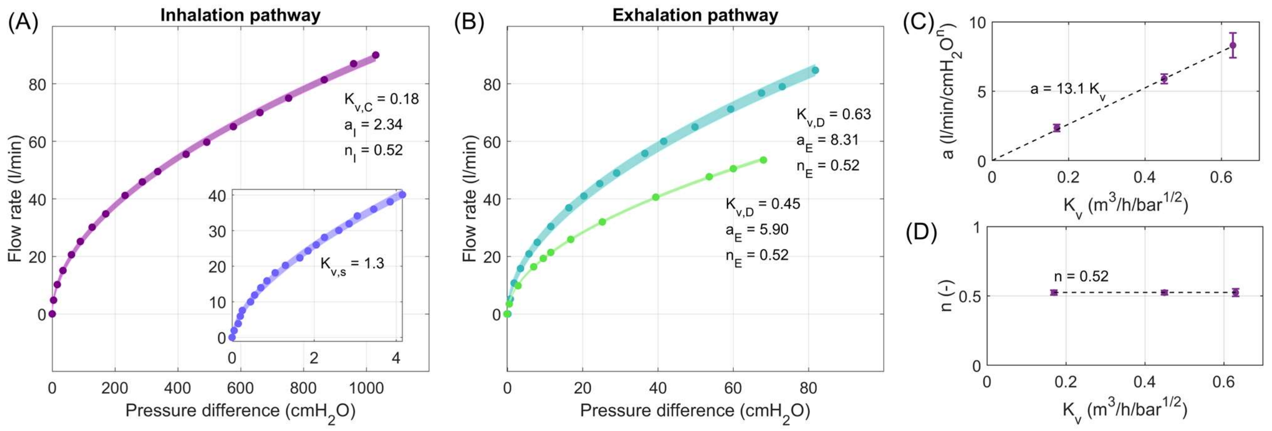

Figure S1: Calibration of the flow-pressure relationship of the inhalation (A) and exhalation pathways (B) and corresponding values applied in Equations 1 and 5 respectively. Inset in panel (A) shows flow-pressure data for calculating $K_{v,s}$ (Equation 2). Shaded regions indicate 95% prediction bounds of the power-law fit. (C) A linear fit through the origin between K_v and a was determined. Error bars represent 95% confidence intervals. (D) No dependence of n on K_v was observed.

Resistance Characterisation

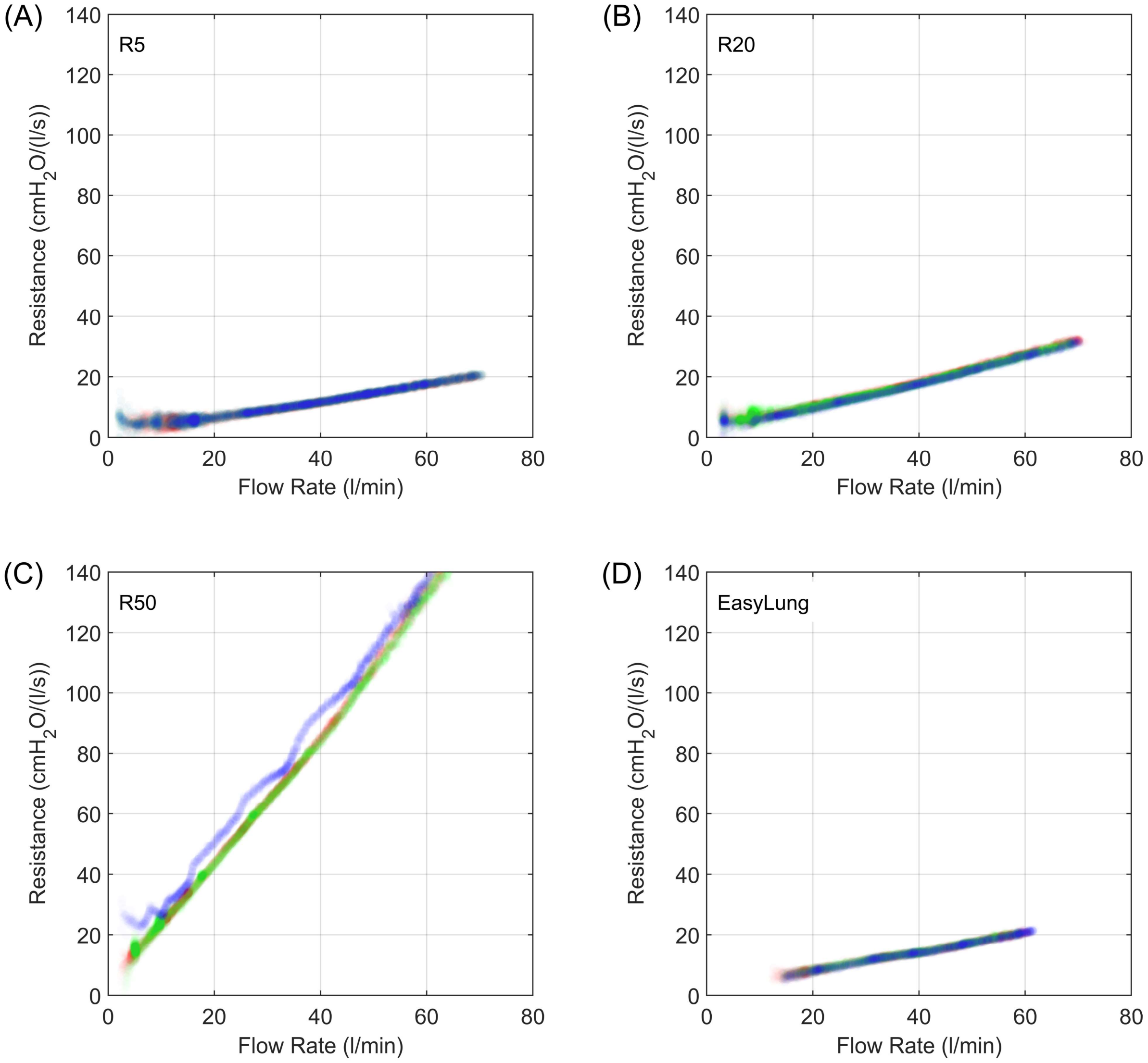

Figure S2: Resistance of the SmartLung as a function of flow rate, rated at (A) 5 cmH2O/(l/s), (B) 20 cmH2O/(l/s), (C) 50 cmH2O/(l/s), and (D) EasyLung rated at 25 cmH2O/(l/s). A custom MATLAB script was implemented to calculate resistance from internal pressure traces recorded by the prototype and external flow rate measurements recorded by a Sensirion SFM3000 series flow meter. Blue, red and green markers represent triplicate measurements for each configuration.

Numerical Modelling Background

Simscape Components

6. Valve C - Inhalation Valve
7. Valve C’ - Inhalation support or relief valve
8. System Pressure Sensor
9. Low Pressure Relief Valve
10. Valve D - Exhalation Valve

Key

Air/Oxygen Mix

Exhaled Breath

Patient Tubing

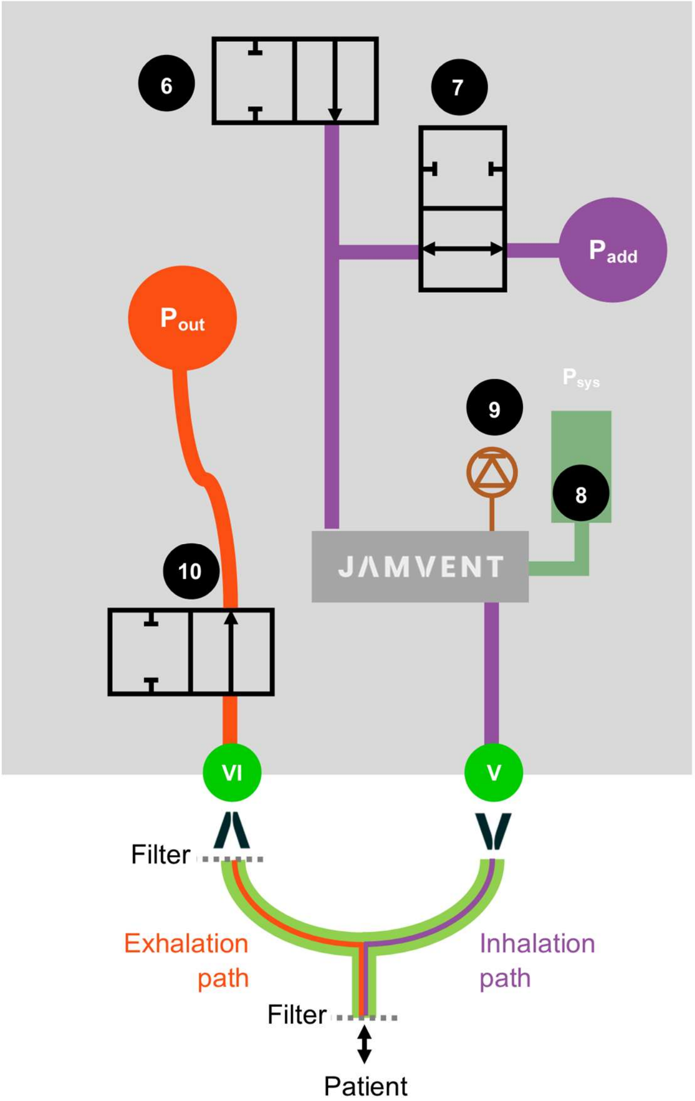

Figure S3: Subsection of the Simscape model showing the patient inflow and outflow pathways. Addition of a support or relief valve, Valve C', in a branch downstream of Valve C, to match the inhalation flow rate to the characteristic obtained from the calibration of the prototype Valve C and its connectors. Adjustment of the downstream pressure of Valve D for the same purpose.

At Valve C, a branch was added downstream with a Valve C’ of the same K_v connected to a pressure source p_{add}, which is calculated as follows. For set pressures p_{res} and p_{sys}, the difference between the expected flow rate in l/min in the experimental circuit and the ISO flow rate through Valve C is

$$\Delta q_I = q_I^{JAM} - q_I^{ISO} = a_I\,(p_{res} - p_{sys})^{n_I} - 395\,K_v^C \sqrt{0.4\left(\frac{p_{res}}{p_{sys}}\right)^2 + 0.6\,\frac{p_{res}}{p_{sys}} - 1} \qquad \text{(S1)}$$

Valve C' should supply, or remove, Δq_I from the circuit, and functions according to the ISO standard, so p_{add} should satisfy

$$\Delta q_I = \varepsilon\,395\,K_v^C \sqrt{0.4\left(\frac{p_{add}}{p_{sys}}\right)^{2\,\varepsilon} + 0.6\left(\frac{p_{add}}{p_{sys}}\right)^{\varepsilon} - 1} \qquad \text{(S2)}$$

In the above equation, ε is the sign of Δq_I, treated as positive for flow directed into the system. This second-degree polynomial in the pressure ratio, of discriminant Δ, has the positive root

$$p_{add} = p_{sys}\left(-\frac{3}{4} + \frac{5}{4}\sqrt{\Delta}\right)^{\varepsilon} \qquad \text{(S3)}$$

At Valve D, the downstream pressure p_{out}, which is normally atmospheric, was adjusted in a similar fashion such that $q_E^{JAM} = q_E^{ISO}$.

ISO Test Traces

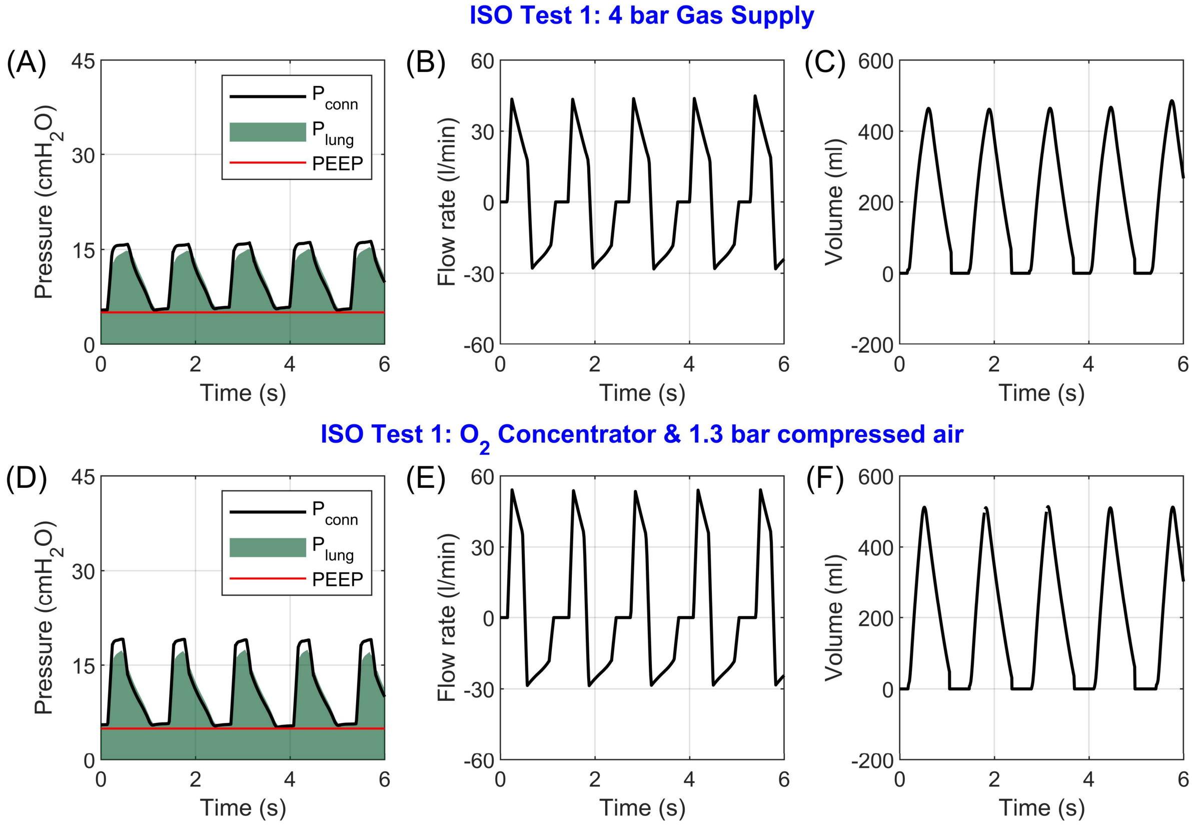

Figure S4 Recorded traces of pressure, flow rate and volume over six seconds for ISO Test 1, when using compressed gas supplies or an oxygen concentrator (related to Figure 7 and Table 2).

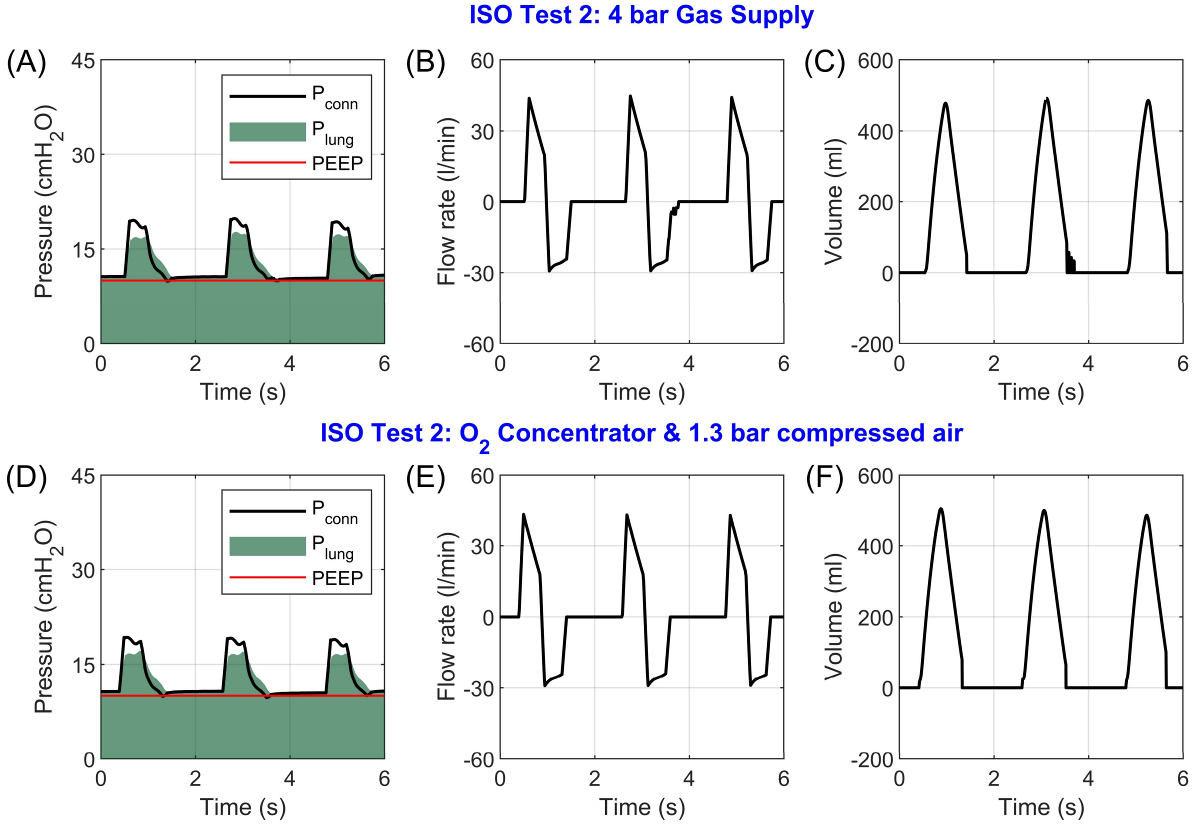

Figure S5: Recorded traces of pressure, flow rate and volume over six seconds for ISO Test 2, when using compressed gas supplies or an oxygen concentrator (related to Figure 7 and Table 2).

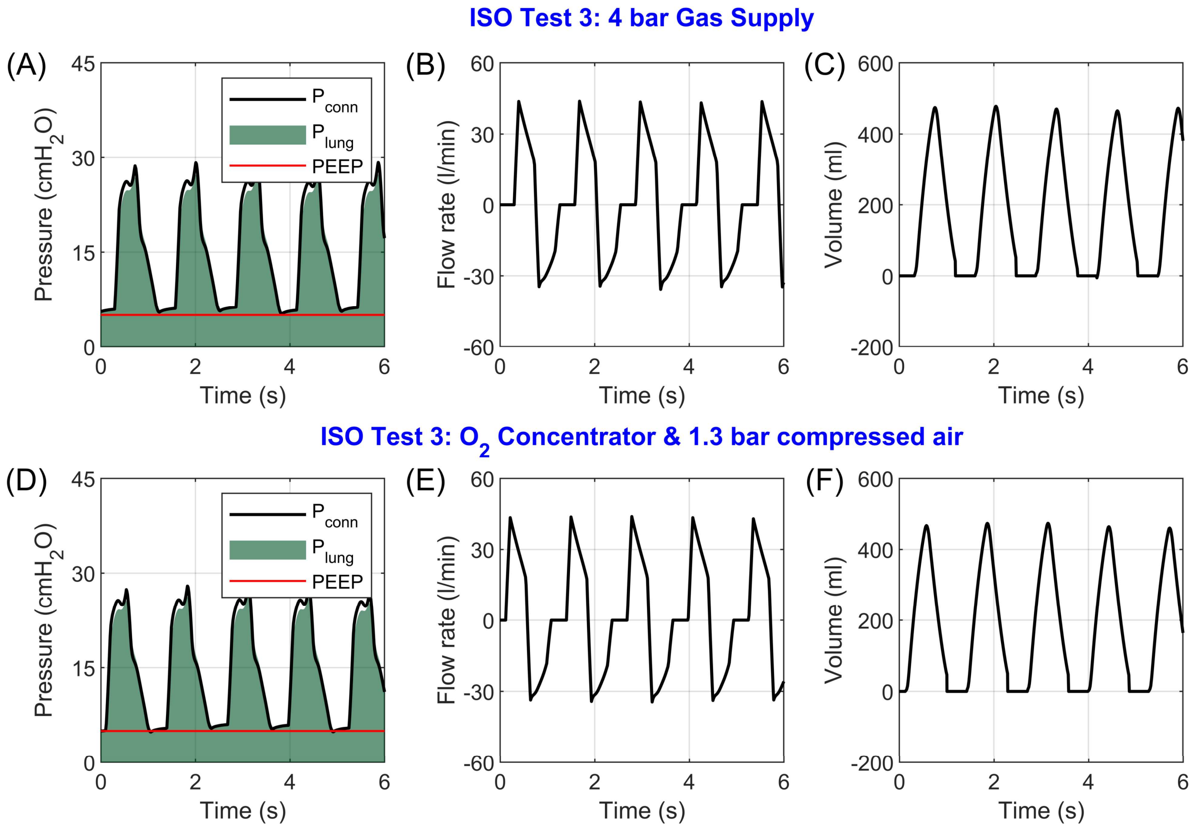

Figure S6: Recorded traces of pressure, flow rate and volume over six seconds for ISO Test 3, when using compressed gas supplies or an oxygen concentrator (related to Figure 7 and Table 2).

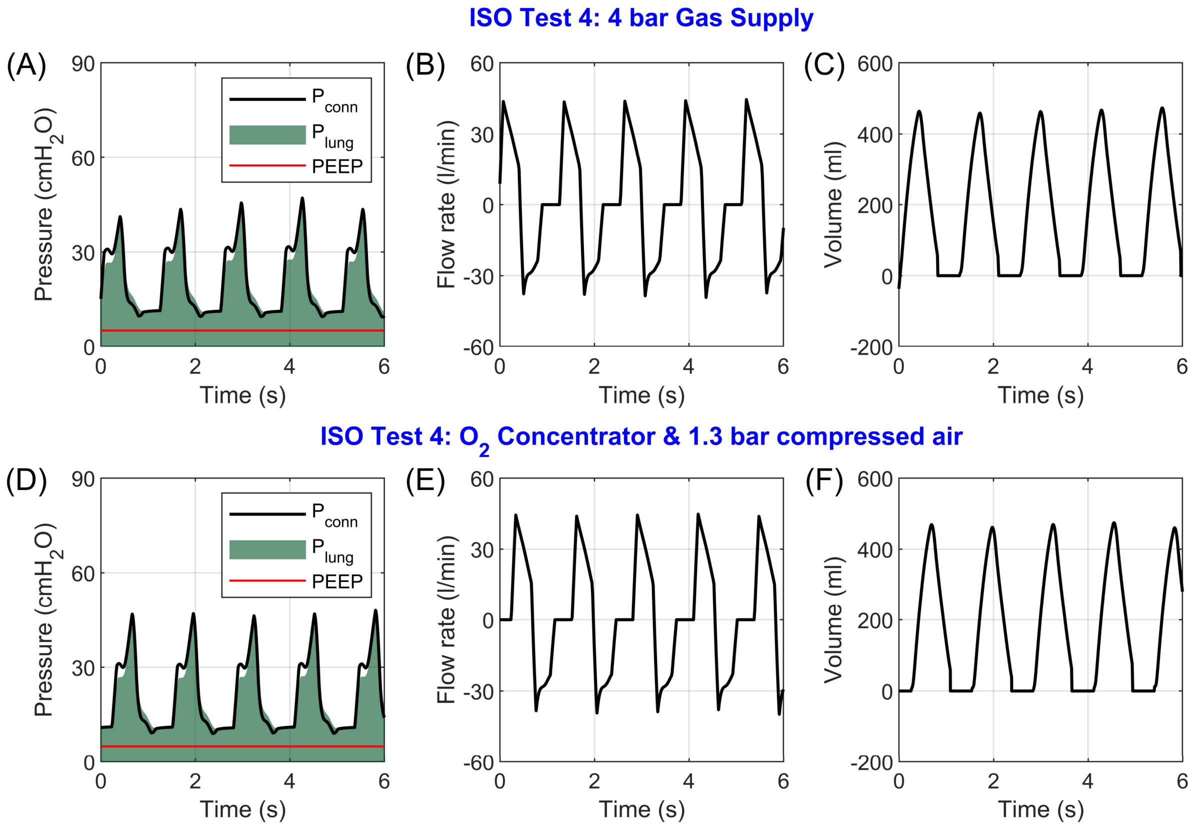

Figure S7: Recorded traces of pressure, flow rate and volume over six seconds for ISO Test 4, when using compressed gas supplies or an oxygen concentrator (related to Figure 7 and Table 2).

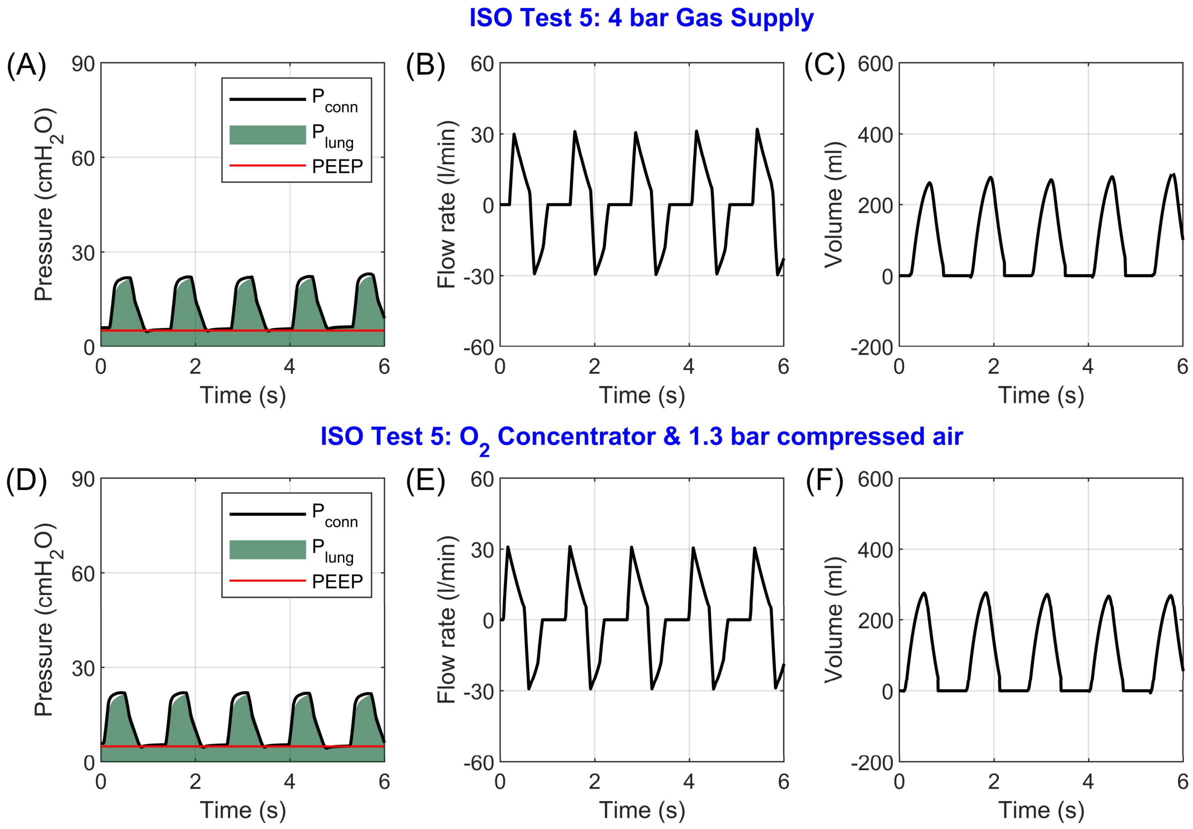

Figure S8: Recorded traces of pressure, flow rate and volume over six seconds for ISO Test 5, when using compressed gas supplies or an oxygen concentrator (related to Figure 7 and Table 2).

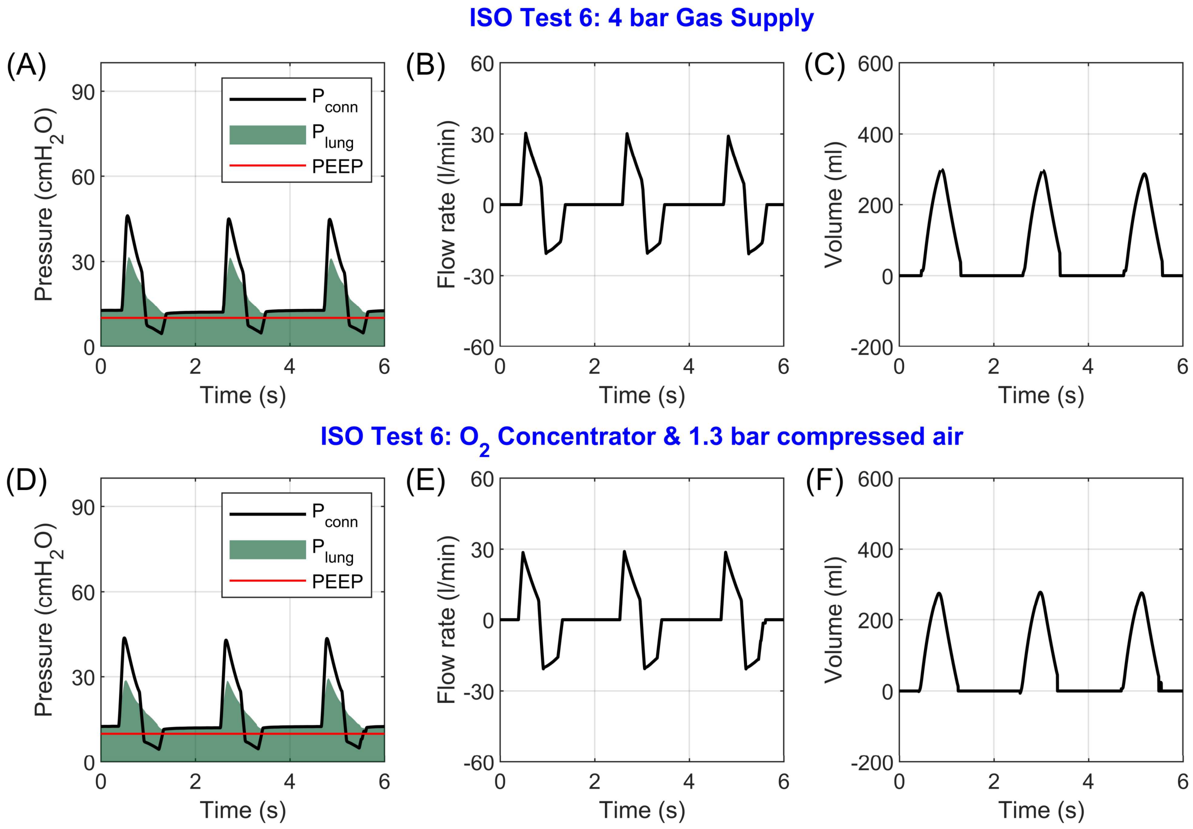

Figure S9: Recorded traces of pressure, flow rate and volume over six seconds for ISO Test 6, when using compressed gas supplies or an oxygen concentrator (related to Figure 7 and Table 2).

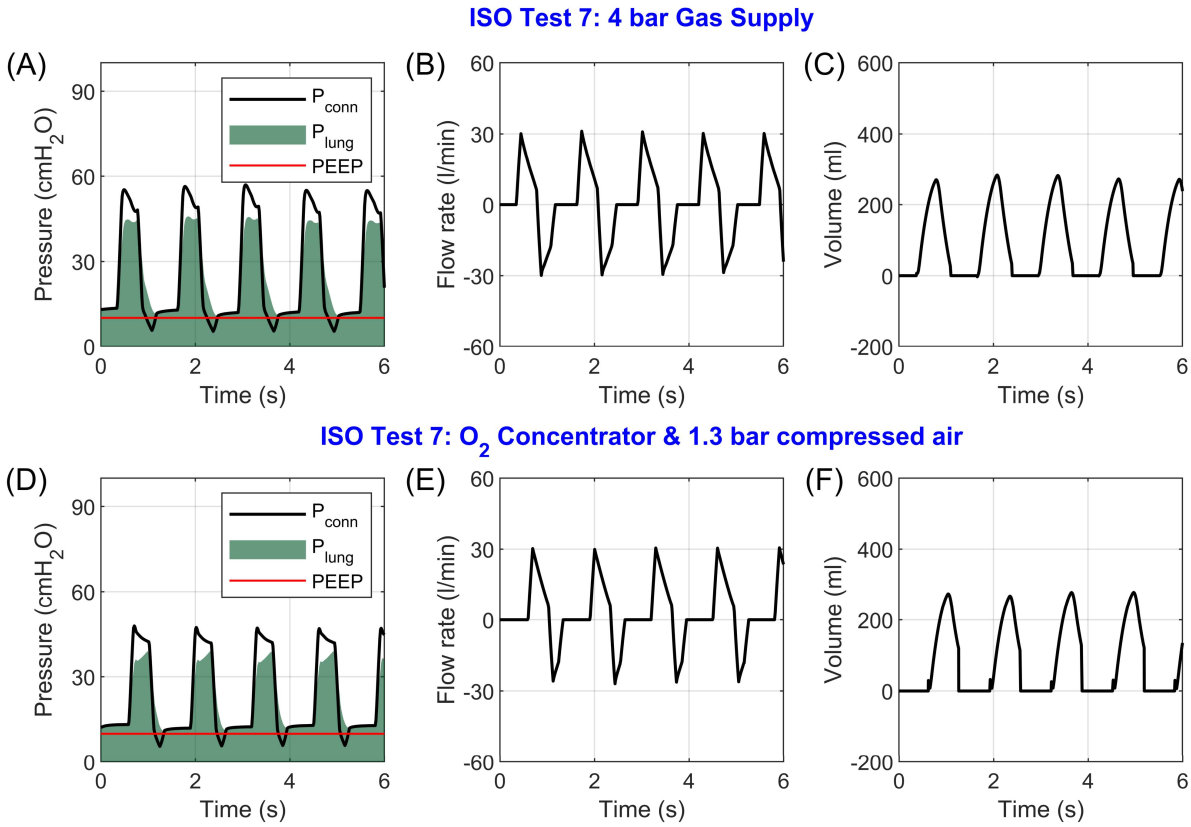

Figure S10: Recorded traces of pressure, flow rate and volume over six seconds for ISO Test 7, when using compressed gas supplies or an oxygen concentrator (related to Figure 7 and Table 2).

Inlet Valve Flow Coefficicents

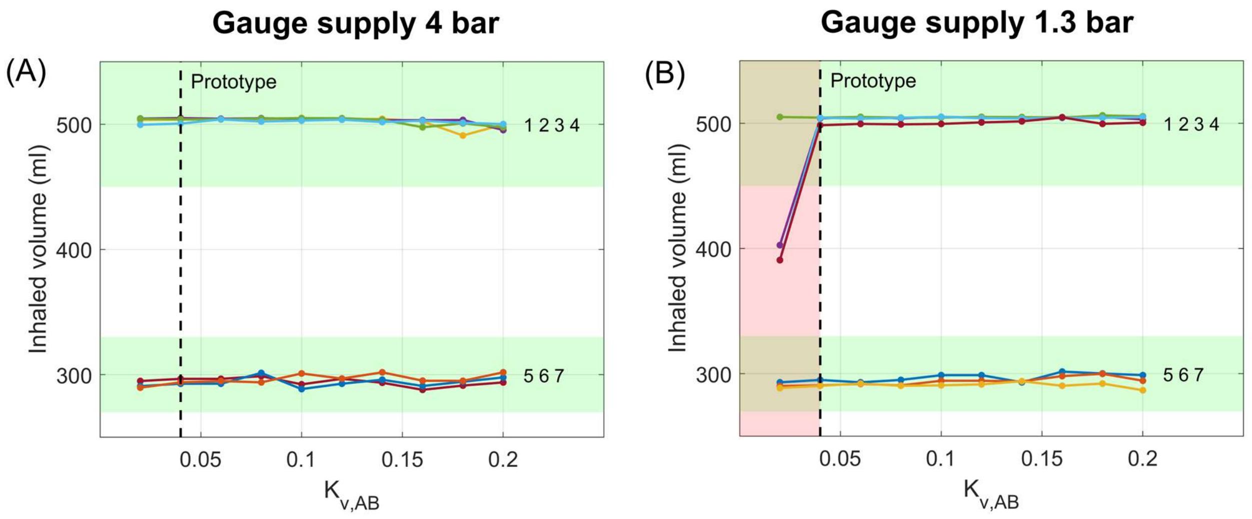

Figure S11: Maintenance of tidal volume for ISO 80601-2-12-2020 cases for $K_{v,AB} \geq 0.02$ at a gas supply pressure of 4 bar (A) and for $K_{v,AB} \geq 0.04$ at a gas supply pressure of 1.3 bar (B).

Effects of Patient Circuit on Flow Rate Calibration

During inhalation: The flow rate is calculated based on P_{sys} and P_{res} (Equation 1), which are both internal, and hence q_I is independent of the patient circuit.

During exhalation: The only resistance that affects the exhaled flow rate, q_E, is R_D (Figure 1B). As there is no flow in the inspiratory branch of the patient tubing during exhalation, the pressure P_{sys} is equal to the value at the Y-piece, downstream of the endotracheal tube, patient HME filter, etc. Hence, the only components that could affect R_D are the patient tubing connecting the Y-piece to the ventilator and the filter on the exhalation port of the ventilator. To investigate how the tubing and filters affect the calibration constants in Equation 5, multiple calibrations were run under various scenarios. The results confirm that the flow rate measurement is robust to different patient circuits (Figure S12).

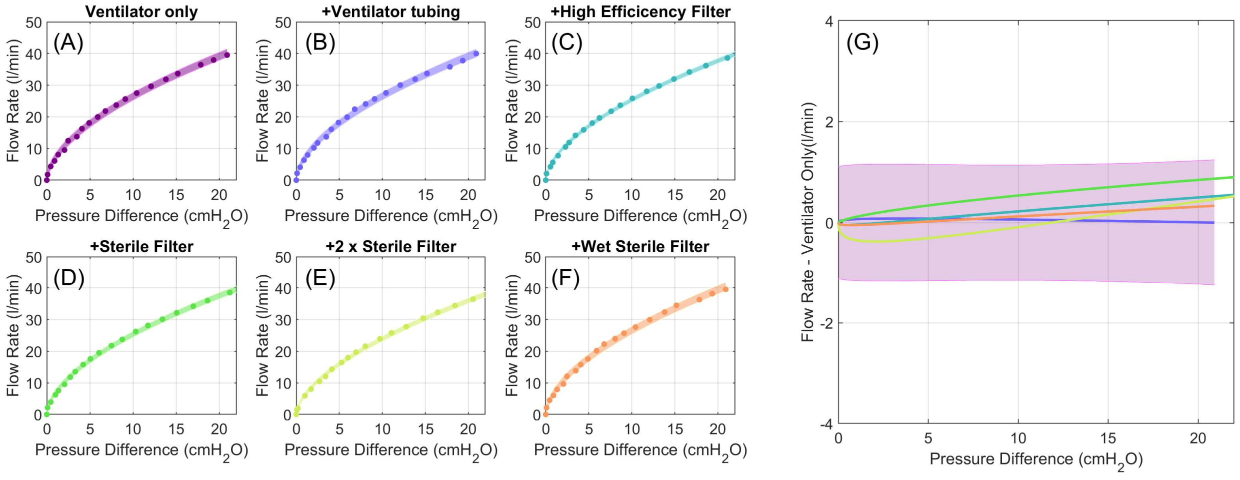

Figure S12: Calibration of the flow-pressure relationship exhalation pathways with various additional components in the system. (A) Ventilator only, (B) 2m of 22mm ID ventilator tubing, (C) A high-efficiency HME filter on the exhalation port (Intersurgical Filta-Therm), (D) A sterile HME filter on the exhalation port (Intersurgical Inter-Therm), (E) 2 sterile HME filters in series on the exhalation port (to provide additional resistance), (F) A wet sterile HME filter on the exhalation port (to mimic a build-up of secretions, although in practice these would be unlikely to pass). (G) Shows the best fit lines of Equation 5 to each case (same colour coding as A-F) and the 95% prediction bounds for the ventilator only case.

Tables

Table S1: For the parametric sweep, 166 cases were conducted out of a possible 243 (see Section 2.3.1 for elimination criteria).

60% FiO_2			
TV	PEEP	RR	E/I
200	5	10	1, 2, 4
200	5	20	1, 2, 4
200	5	35	1, 2, 4
200	10	10	1, 2 4
200	10	20	1, 2, 4
200	10	35	1, 2, 4
200	15	10	1, 2, 4
200	15	20	1, 2, 4
200	15	35	1, 2, 4
400	5	10	1, 2, 4
400	5	20	1, 2, 4
400	5	35	1, 2, 4
400	10	10	1, 2, 4
400	10	20	1, 2, 4
400	10	35	1, 2
400	15	10	1, 2, 4
400	15	20	1, 2
400	15	35	1
600	5	10	1, 2
600	5	20	1, 2

75% FiO_2			
TV	PEEP	RR	E/I
200	5	10	1, 2, 4
200	5	20	1, 2, 4
200	5	35	1, 2, 4
200	10	10	1, 2, 4
200	10	20	1, 2, 4
200	10	35	1, 2
200	15	10	1, 2
200	15	20	1, 2, 4
200	15	35	1, 2, 4
400	5	10	1, 2, 4
400	5	20	1, 2, 4
400	5	35	1, 2
400	10	10	1, 2, 4
400	10	20	1, 2, 4
400	10	35	1, 2
400	15	10	1, 2, 4
400	15	20	1, 2, 4
400	15	35	1, 2
600	5	10	1, 2, 4
600	5	20	1, 2
600	10	10	1

95% FiO_2			
TV	PEEP	RR	E/I
200	5	10	1, 2, 4
200	5	20	1, 2, 4
200	5	35	1, 2, 4
200	10	10	1, 2, 4
200	10	20	1, 2, 4
200	10	35	1, 2, 4
200	15	10	1, 2, 4
200	15	20	1, 2, 4
200	15	35	1, 2, 4
400	5	10	1, 2, 4
400	5	20	1, 2, 4
400	5	35	1
400	10	10	1, 2, 4
400	10	20	1, 2, 4
400	10	35	1, 2
400	15	10	1, 2, 4
400	15	20	1, 2, 4
400	15	35	1, 2
600	5	10	1, 2, 4
600	5	20	1, 2
600	10	10	1, 2

Table S2: Statistics for Bland-Altman Analysis comparing prototype and flow analyser (see Figure 5). T-test evaluates null hypothesis that the two measurement methods have no average offset. Correlation coefficient evaluates whether any offset scales with the value measured.

**Clinical parameter**	**Mean Difference**	**2SD range (lower, upper)**	**t-test p-value**	R^2
V_T (ml)	-7	-36, 22	<0.001	0.03
RR (bpm)	-0.01	-0.2, 0.2	0.04	0.005
PEEP (cmH_2O)	-0.6	-1.4, 0.2	<0.001	0.03
FiO_2 (%)	-2	-7, 3	<0.001	0.2

Table S3: Statistics for Parametric analysis (see Figure 6). T-test evaluates null hypothesis that the average parameter values were equal to the target values. Correlation coefficient evaluates whether any offset scales with the value measured.

Clinical parameter	Mean Difference	2SD range	t-test p-value	R^2
V_T (ml)	-17	-25, 12	<0.001	0.13
RR (bpm)	-0.2	-1.0, 0.6	<0.001	0.02
PEEP (cmH_2O)	1.0	-0.3, 2.3	<0.001	0.04
FiO_2 (%)	-1.3	-3.7, 1.1	<0.001	0.08

Table S4: Statistics for ISO tests (see Figure 7).

	4 bar Gas Supply		O_2 Concentrator & 1.3 bar compressed air	
Clinical parameter	Mean Difference	2SD range	Mean Difference	2SD range
V_T (ml)	8	-9, 26	15	-22, 52
PEEP (cmH_2O)	1.1	-0.4, 2.7	1.2	-0.4, 2.7
FiO_2 (%)	1.9	0.7, 3.1	0.92	-1.1, 2.9

Table S5: Statistics for Durability analysis (see Figure 8). Correlation coefficient evaluates whether the performance varied in time.

Clinical parameter	Mean Value	2SD range	R^2
V_T (ml)	407	399, 415	0.01
PEEP (cmH_2O)	5.6	4.9, 6.3	0.02

Table S6: Statistics for Bland-Altman Analysis comparing prototype and numerical model (see Figure 10).

Clinical parameter	Mean Difference	2SD range	t-test p-value	R^2	Correlation p-value
V_T (ml)	-8	-30,14	0.03	0.2	< 0.01
PEEP (cmH_2O)	-0.4	-1.0,0.2	< 0.01	0.3	< 0.001
PiP (cmH_2O)	-0.2	-6.8,6.4	0.9	0.002	0.8
T_{ex}^* (-)	0.02	-0.03, 0.05	< 0.05	0.001	0.8
